# Experimental investigation of tunnel fire spread under moving fire source conditions

**DOI:** 10.1371/journal.pone.0336712

**Published:** 2026-02-24

**Authors:** Tong Wang, Yi Qin, Zhihao Zhang, Yue Xiang, Xiong Zhu, Tao Fan

**Affiliations:** 1 School of Safety Science and Engineering, Chongqing University of Science & Technology, Chongqing, China,; 2 Key Laboratory of Gas and Fire Control for Coal Mines, China University of Mining and Technology, Xuzhou, Jiangsu, China; 3 China Academy of Safety Science and Technology, Beijing, China; 4 Nanchong Vocational College of Science and Technology, Nanchong, Sichuan, China; 5 The Eleventh Engineering Bureau of Hydropower and Water Resources, Zhengzhou, Henan, China; University of Salerno: Universita degli Studi di Salerno, ITALY

## Abstract

Fires caused by moving fire sources are more complex and hazardous than those initiated by stationary sources. In tunnel fire scenarios, ignition sources often vary in velocity and elevation, which markedly affect fire dynamics. A small-scale experimental platform was employed to examine the impact of fire source height and speed on flame behavior, smoke propagation, and temperature distribution in tunnel fires. The experimental results demonstrate that the critical velocity associated with noticeable flame attenuation exhibits a distinct non-monotonic dependence on fire-source elevation. Specifically, as the height increases from 7.5 cm to 10 cm, the critical velocity rises from 0.3 m/s to 0.8 m/s, yet decreases to 0.5 m/s at 12.5 cm. At a fixed height, increasing the movement speed from 0.1 m/s to 1.0 m/s reduces flame height by up to 40% and increases the flame tilt angle by more than 50%. Conversely, at a constant speed, flame tilt angle first decreases and then increases with increasing fire source height, while flame height initially rises and then falls. A clear quantitative relationship is identified between flame height and movement speed; specifically, the ratio of flame height (L) to effective tunnel height (H) shows a distinct functional relationship with $(Q^{1/3}H^{1/3}!/v$. Moreover, Smoke layer thickness decreases with increasing fire source height. The reverse smoke flow length increases and then decreases with increasing fire source speed. Ceiling temperatures show a pronounced dependence on movement speed: at 0.1 m/s, the peak temperature reaches 89.4°C with a height-induced variation of approximately 20°C, whereas at 1.0 m/s, the peak drops to 37°C and the temperature difference narrows to about 2°C.

## 1. Introduction

With rapid economic development and accelerating urbanization, tunnels—critical components of transportation infrastructure—are constructed and operated on an increasingly large scale [1]. Owing to their enclosed and elongated geometry, as well as limited emergency exits, tunnel fires pose significant threats to both human life and structural integrity, as the evacuation of high-temperature smoke is often inefficient [2,3]. Unlike fires caused by stationary sources, tunnel fires frequently involve moving fire sources due to vehicle mobility, resulting in markedly more complex fire dynamics [4,5]. In practice, tunnel fires may be triggered by various ignition scenarios, such as tire combustion, engine failure, spontaneous ignition of cargo, or hazardous material leakage [6,7]. These ignition modes introduce variability in the vertical position of the fire source—that is, the fire source height. Moreover, since vehicles are typically the main source of ignition in tunnel fires, their movement adds another layer of variability in the form of differing fire source speeds. Consequently, moving fire sources in tunnels are characterized by coupled variations in both height and velocity.

Recent studies have investigated the characteristics of smoke propagation and temperature distribution in tunnel fires under various conditions. Tang et al. employed the Fire Dynamics Simulator (FDS) to examine the effect of vehicle height on smoke spread and extraction in a cross-river tunnel. Their findings indicated that an increase in fire source height intensified flow field turbulence downstream, thereby reducing the extent of smoke spread [8]. Qiu et al. conducted scaled model experiments to analyze the maximum ceiling temperature and lateral temperature profiles under dual fire source scenarios, and subsequently proposed a predictive model for these thermal characteristics [9]. Through tunnel model tests involving varying widths and heights, Li et al. found that under well-ventilated conditions, the fuel heat release rate was largely unaffected by tunnel width, height, or ventilation speed [10]. Tao et al. utilized computational fluid dynamics (CFD) simulations to study full-scale tunnel fires and observed that fire source height had a notable influence on ceiling temperature distribution. Specifically, an elevated fire source led to a slower temperature decay near the ceiling, and at certain heights, the smoke mass flow rate was markedly lower than that associated with ground-level fires. However, as fire power increased, this difference gradually diminished [11]. Wang et al. investigated fire behavior using small-scale tunnel models of varying lengths and found that in longer tunnels with natural ventilation, the smoke layer settled, obstructing fresh air inflow and causing fire self-extinction. In contrast, shorter tunnels under identical conditions exhibited no smoke settlement or self-extinction [12]. Liu et al. examined the effect of fire source elevation on tunnel fire development and observed that increasing the elevation enhanced the fuel combustion rate and shifted the location of peak ceiling temperature [13]. More recently, studies have further highlighted that fire source elevation can significantly affect critical ventilation velocity and smoke back-layering characteristics under longitudinal ventilation, reinforcing the importance of height-related effects in tunnel fire dynamics [14]. Han et al., using small-scale pool fire models, reported that combustion rate increased with fire source height, and this increase became more pronounced with larger fire source areas [15]. Luo performed scale-model fire experiments under varying fire source heights and found that, under longitudinal ventilation with an off-center fire source, flame height first increased and then decreased as the effective ceiling height declined. Meanwhile, flame tilt angle initially decreased and then increased with increasing fire source height [16]. Luo also revised previous models and developed a new lateral temperature distribution model that simultaneously incorporates the effects of longitudinal airflow velocity and fire source elevation.

The movement of the fire source markedly affects flame morphology, smoke propagation, and related fire dynamics. Numerous researchers have investigated tunnel fire behavior under moving fire source conditions. Lou et al. conducted simulations using Fluent and discovered that tangential forces generated by velocity and acceleration, combined with buoyant forces from combustion, altered the fractal characteristics of moving flames, making them more complex than those of stationary flames—an effect that cannot be fully explained by the concept of relative motion alone [17,18]. Wang et al. performed model experiments to analyze airflow velocity and smoke concentration distributions in a tunnel following a train fire, comparing scenarios with moving and stationary fire sources [19]. Wei et al. used FDS simulations and small-scale experiments to study tunnel fires involving moving sources. Their results indicated that fire source motion caused smoke to spread predominantly upstream, while the flame lagged behind, thereby reducing the temperature beneath the tunnel ceiling [20]. Fu utilized FDS to construct a tunnel fire model for a comprehensive analysis of temperature and smoke behavior. The study revealed that the fire jet collided with the ceiling at shifted locations due to source movement and identified a critical velocity of 3 m/s, beyond which smoke backflow was eliminated [21]. Li et al. compared the combustion characteristics of moving fire sources under natural ventilation with stationary fire sources under longitudinal ventilation through scaled tunnel experiments. They found that when the movement speed matched the ventilation speed, the heat release rate of the moving source was reduced, resulting in a lower maximum ceiling temperature and a shorter duration of elevated temperatures [22]. Liu et al. applied a three-dimensional dynamic grid model to study tunnel fire propagation with moving fire sources, concluding that the rate of ceiling temperature rise increased with both fire source intensity and blockage ratio [23]. Deng et al. used FDS to explore smoke propagation under varying movement speeds and observed that moving fire sources disrupted the thermal circulation mechanism between ceiling jet flow and cold air to some extent [4]. An et al. conducted simulations of tunnel fires with moving sources under natural ventilation using Fluent, revealing that source movement disrupted ceiling jet-induced circulation and generated swirling turbulent flows downstream of the fire source [24,25]. Recent numerical studies focusing on subway and train fire scenarios have further demonstrated that source movement speed plays a critical role in smoke spread patterns, back-layering behavior, and emergency operating strategies, emphasizing the importance of movement-induced effects in tunnel fire safety analysis [26]. Lu employed Fluent to study the effects of different fire source velocities on smoke dispersion, temperature distribution, and tunnel air pressure in highway tunnels. The findings indicated that with increasing source speed, the smoke backflow length increased, the distance between the fire source and peak ceiling temperature extended, the maximum ceiling temperature continuously decreased, and the total smoke layer length initially increased and then decreased [27].

In summary, changes in fire source height and movement speed have non-negligible impacts on the ceiling temperature distribution and smoke diffusion patterns of tunnel fires. In addition, previous studies have shown that flame–ceiling interactions, including radiative heat transfer, can further influence the thermal response of tunnel ceilings under fire conditions [28]. Moreover, tunnel fires caused by moving fire sources and stationary fire sources differ markedly in terms of heat release rate and smoke layer development. Therefore, the coupling effect of different fire source heights and speeds may produce an influence mechanism different from that under the change of a single condition. Meanwhile, in actual tunnel fires, fire sources may have different heights and movement speeds, but current studies on fire source height mainly focus on stationary fire sources. Research on the impact of moving fire source height on tunnel fires is relatively scarce, especially the lack of systematic studies on the evolution laws of tunnel fires under the coupling of different fire source heights and movement speeds. To address this gap, the present study employs a small-scale tunnel model to systematically investigate flame morphology, temperature distribution, and smoke propagation associated with moving fire sources at different elevations and velocities. The primary objective is to elucidate how variations in fire source height and movement speed jointly influence combustion behavior, smoke dispersion characteristics, and thermal conditions within the tunnel. The findings are intended to contribute to an improved physical understanding of tunnel fires involving moving fire sources, thereby providing useful insight for fire safety analysis and control strategies. It should be emphasized that the present work is conducted as an exploratory, mechanism-oriented investigation focusing on the macroscopic effects of fire source movement speed and elevation on tunnel fire behavior. Accordingly, the conclusions are interpreted in terms of physical trends and dominant flow–flame interaction mechanisms, rather than as direct quantitative extrapolations to full-scale tunnel fire scenarios.

## 2. Materials and methods

### 2.1. Experimental platform

In tunnel fire research, maintaining a consistent Froude number—which characterizes the ratio of inertial to gravitational forces—is a widely adopted approach to achieve dynamic similarity between model-scale and full-scale scenarios [29–33]. In this study, an existing small-scale experimental model was utilized, designed according to the Froude similarity criterion to accurately replicate the physical behavior of full-scale tunnel fires [34]. The model parameters, including the geometric scaling factor, are summarized in Table 1.

**Table 1:** Proportional coefficient statics [34].

Physical Parameter	Scaling Laws
Heat Release Rate Q (kW)	$\frac{Q_{F}}{Q_{M}}={\left(\frac{l_{F}}{l_{M}}\right)}^{5/2}$
Velocity v (m/s)	$\frac{v_{F}}{v_{M}}={\left(\frac{l_{F}}{l_{M}}\right)}^{1/2}$
Time t (s)	$\frac{t_{F}}{t_{M}}={\left(\frac{l_{F}}{l_{M}}\right)}^{1/2}$
Temperature T (K)	$T_{F}=T_{M}$

**Fig 1:**
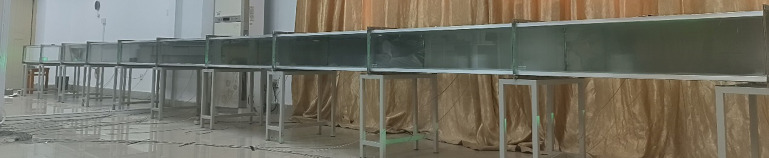
Experimental model diagram.

The experimental model measured 10 m in length, 0.5 m in width, and 0.25 m in height, corresponding to a geometric scale ratio of 1:20. Transparent fire-resistant glass panels were installed on the front and rear sides to serve as observation windows, while the remaining sides were constructed using fireproof insulating panels to ensure thermal containment [34]. A schematic of the model setup is provided in Fig 1.

**Fig 2:**
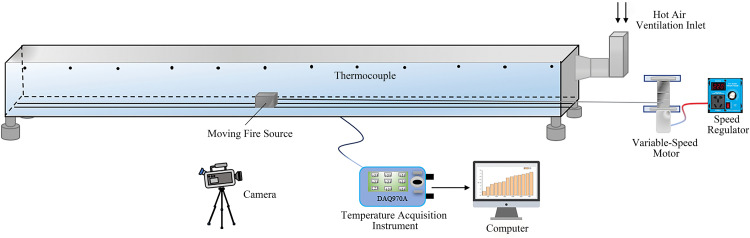
Schematic diagram of the equipment layout.

The layout of the experimental apparatus is illustrated in Fig 2. A laser emitter was positioned at the far end of the tunnel, while a camera was installed directly in front of the tunnel to capture the smoke propagation length and layer thickness during the experiments.

The fire source is driven by a speed-regulating motor to move at a constant speed. 100 g of high-calorific mineral oil was selected as a surrogate fuel, as the macroscopic heat release behavior governing buoyancy-driven fire dynamics is largely independent of the detailed chemical composition of actual vehicle combustibles under Froude-scaled conditions. The combustion efficiency of the mineral oil was 0.75, and its specific heat of combustion (ΔH) was 46.36 MJ/kg. In the experiment, and a separate experiment for measuring the fuel mass loss rate was conducted before the formal experiment: during the combustion process, real-time monitoring of the fuel’s mass loss was performed using an electronic balance (model: Explorer EX10202ZH, precision: 0.01 g); subsequently, based on the law of mass change over time, the average mass loss rate of combustion was calculated to be approximately 0.1 g/s. Based on Eq. (1), the corresponding heat release rate (*HRR*) of the fire source is calculated to be 5 kW. The heat release rate (HRR) of the fire source was set to 5 kW. According to Froude scaling laws, this corresponds to approximately 8.9 MW at full scale. Based on relevant literature, this value represents a fire scenario involving a large passenger car or a small van [35].


$$Q=\mu \dot{m}H_{c}$$ (1)

where: *Q*——Heat release rate of the fire source, kW: *μ*——Fuel combustion efficiency; $\dot{m}$——Fuel mass loss rate, g/s; *H*_*c*_——Calorific value of the fuel, kJ/g;

To enhance smoke visibility, given the low optical density of the smoke produced by mineral oil combustion, a smoke-generating cake (smoke production rate: 25 mg/m^3^) was introduced to provide tracer smoke for flow visualization. The smoke cake functioned solely as a passive tracer and exhibited negligible heat release and mass flux compared with the primary fire source and the entrained airflow; therefore, its introduction does not significantly affect the smoke dynamics or ceiling temperature distribution.

**Fig 3:**
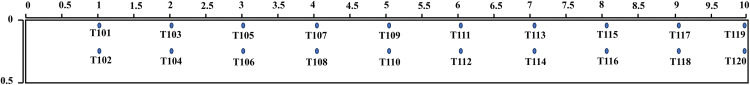
Distribution map of the temperature measurement points of the ceiling.

To ensure experimental accuracy, a Model KA12 anemometer was used to measure indoor wind speed and direction before testing, confirming that the experiments were conducted under conditions with minimal wind speed fluctuations. To accurately characterize the macroscopic longitudinal attenuation of the smoke temperature field, twenty K-type thermocouples were installed along the tunnel centerline at intervals of 1.0 m, with reference to relevant literature [23]. As shown in Fig 3, the sensors were distributed into two parallel arrays: the central array (T102, T104,..., T120) aligned along the tunnel centerline, and the lateral array (T101, T103,..., T119) positioned 5 cm from the right sidewall. Distinct from stationary fire scenarios, the moving fire source creates a continuous scanning mechanism relative to the fixed sensors. The dynamic variation in the position of the fire source, combined with high-frequency data acquisition, effectively translates high temporal resolution into high spatial resolution. This approach ensures the precise capture of peak temperatures and the comprehensive registration of global attenuation trends.

### 2.2. Experimental condition settings

Considering that vehicles in the tunnel may exhibit different moving speeds due to factors such as vehicle damage and active driver intervention after fire ignition, six different moving speed operating conditions were set in this study with reference to relevant research findings of previous studies [22,36–37]: 0.1 m/s, 0.15 m/s, 0.3 m/s, 0.5 m/s, 0.8 m/s, and 1.0 m/s.

Statistical data indicate that the typical height of passenger cars ranges from 1.4 to 1.9 m, while that of trucks spans from 2.2 to 4.2 m [11]. However, in realistic tunnel fire scenarios, the effective fire source height and combustion surface are generally lower than the total vehicle height. Consequently, the actual effective fire source height is estimated to fall within the range of 0 to 3.0 m. Based on the 1:20 geometric scaling ratio, the corresponding height range in the model is 0–15 cm. Considering that ground-level fires have been widely investigated in previous studies, this research focuses specifically on elevated fire sources. To systematically cover the low, medium, and high levels within this effective range, and with reference to height settings in previous literature [11], three representative fire source heights were selected: 7.5 cm (representing the lower boundary of a cargo fire or the roof of a car), 10 cm (representing the cargo center), and 12.5 cm (representing the upper cargo section).

The experimental ambient temperature was maintained stable by a constant temperature control device, and all experiments were conducted under the conditions of an ambient temperature of 25°C and natural ventilation. To ensure data reliability, each condition was tested four times, and the average values were used for analysis. The specific experimental conditions are summarized in Table 2.

**Table 2:** Experimental working conditions.

Operating Condition	Fire Source Height(cm)	Fire Source Movement Speed(m/s)
1	7.5	0.1
2		0.15
3		0.3
4		0.5
5		0.8
6		1
7	10	0.1
8		0.15
9		0.3
10		0.5
11		0.8
12		1
13	12.5	0.1
14		0.15
15		0.3
16		0.5
17		0.8
18		1

### 2.3. Research procedure

**Fig 4:**
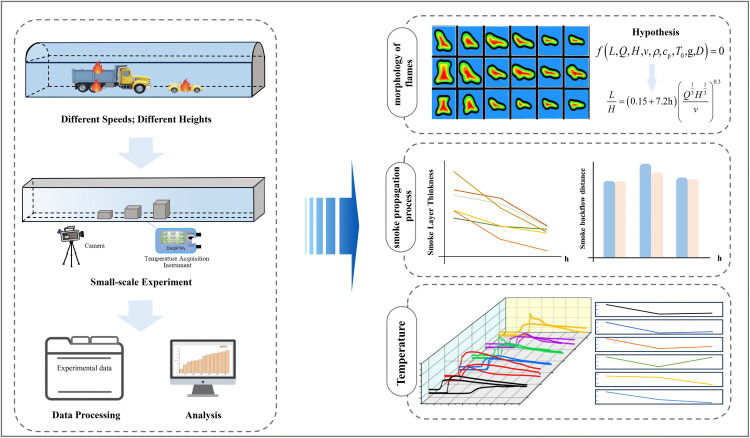
Flowchart of the experimental research methodology.

To systematically investigate the coupling effects of fire source height and movement speed on tunnel fire dynamics, a rigorous experimental protocol was established. First, experiments were conducted on a reduced-scale tunnel platform constructed based on Froude similarity criterion, under a matrix of operating conditions with varying fire source heights (7.5, 10, and 12.5 cm) and movement velocities (0.1–1.0 m/s). During the tests, a temperature acquisition system was employed to record the longitudinal ceiling temperature profiles, while a high-definition camera system simultaneously captured the transient flame morphology and smoke propagation characteristics. Subsequent analysis of the experimental data elucidated the influence of fire source height and movement speed on flame geometry, smoke layer thickness, and temperature evolution. The overall research flowchart is illustrated in Fig 4.

## 3. Results and discussions

### 3.1. Analysis of changes in the morphology of moving flames

**Fig 5:**
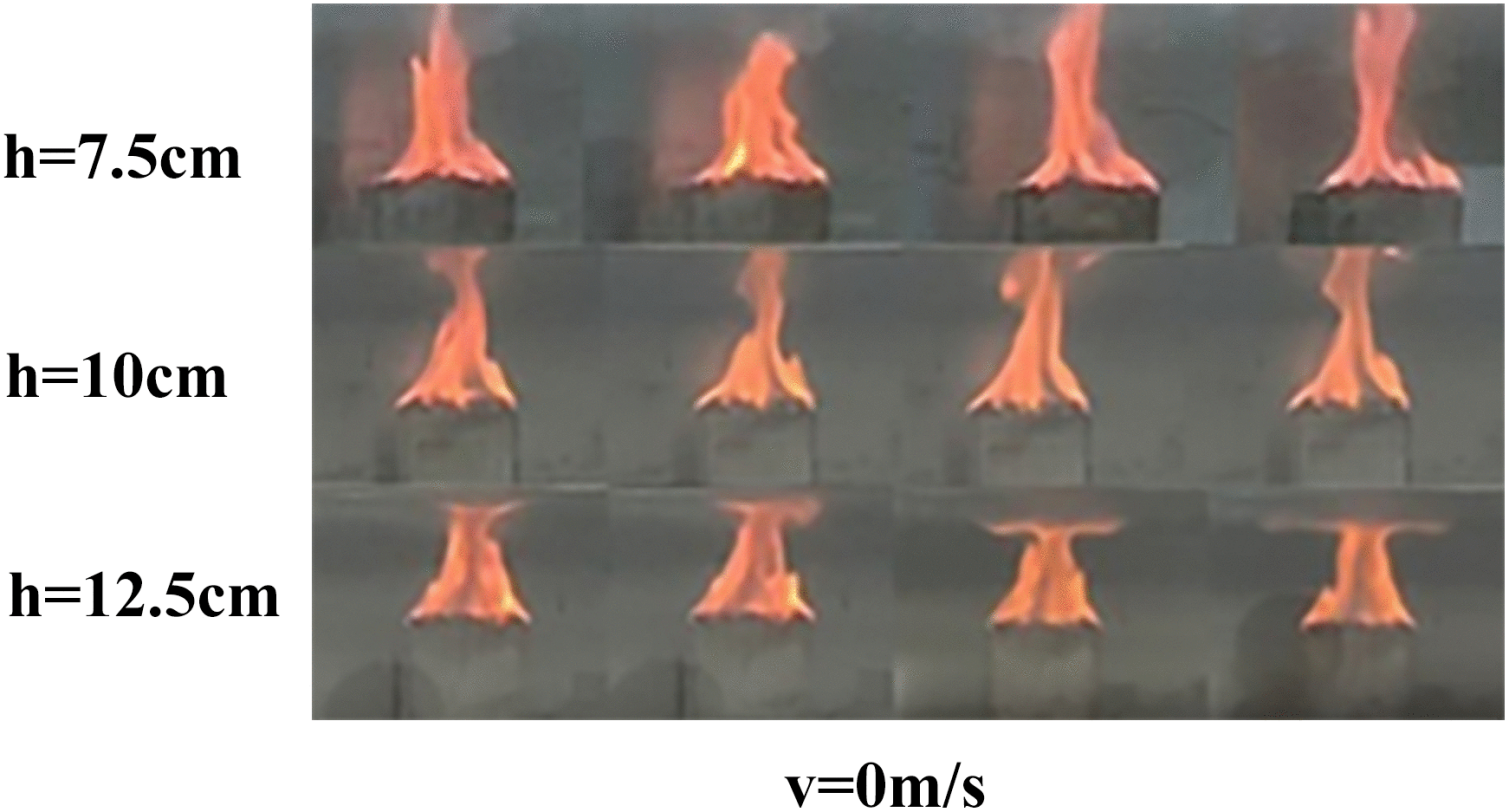
Part of the flame combustion form under working conditions of stationary fire source.

**Fig 6:**
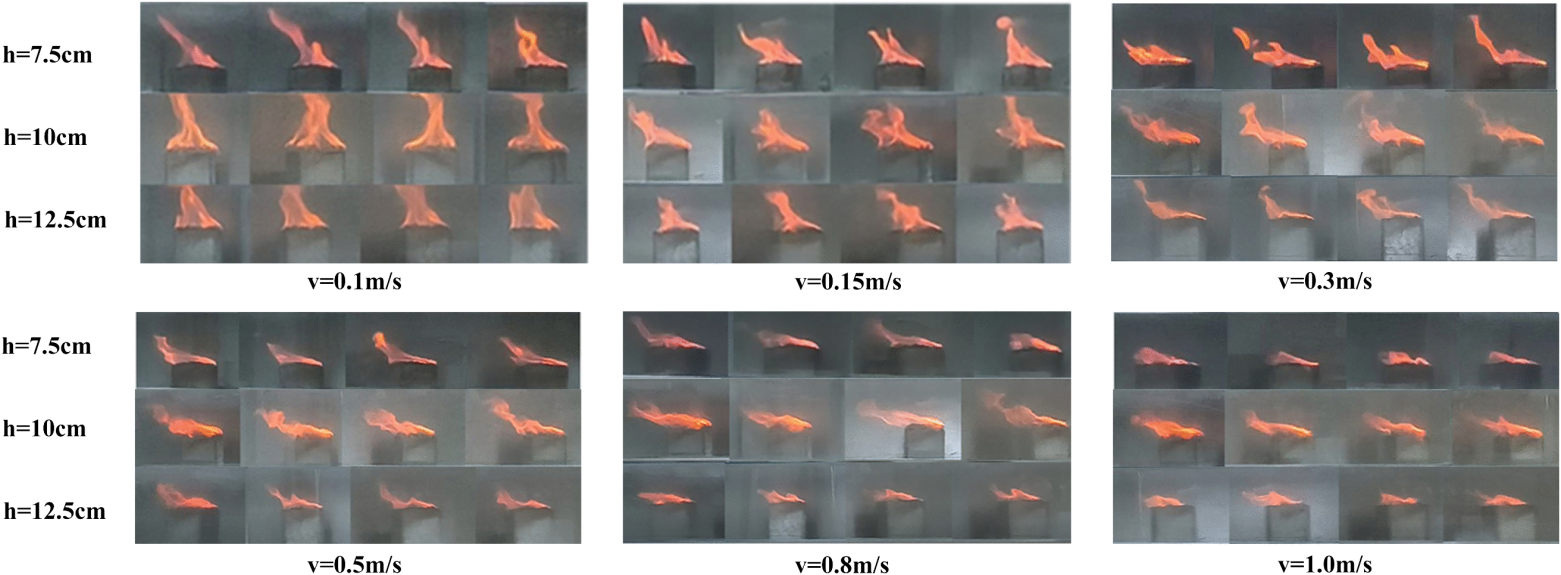
Part of the flame combustion form under various working conditions of the mobile fire source.

The flame is a fundamental indicator of the combustion process, as its shape directly reflects the combustion state of the fire source and the extent of its thermal interaction with the surrounding air. Compared to stationary fire sources, moving fire sources exhibit markedly different flame morphologies. Representative flame shapes observed under various experimental conditions are presented in Figs 5 and 6.

To further investigate the influence of fire source movement speed and height on flame morphology, flame probability contour maps were generated in this study with reference to previous experimental methods [38–40]. In the experiment, a high-definition camera was fixed directly in front of the center of the tunnel scaled model to minimize perspective distortion. The video was recorded at 30 fps, a frame rate chosen to provide sufficient temporal resolution to capture the characteristic puffing frequency of the flame and minimize frame rate influence on the statistical results. Camera calibration was strictly performed by using the known characteristic dimensions of the burner as a spatial reference to establish the precise pixel-to-meter conversion ratio. The specific procedures were as follows: For each working condition, a video segment where the fire source was in the central shooting area and moved a distance of 1 meter was selected. This selection helps eliminate perspective deviations caused by excessive displacement. Each exported frame was sequentially converted into a grayscale image and processed using Otsu’s adaptive thresholding method in MATLAB to ensure robust flame separation. Finally, all binarized images were subjected to a superposition operation to calculate the appearance frequency at each pixel, and the flame probability contour maps under each working condition were plotted using Tecplot. The resulting diagrams are presented in Fig 6.

**Fig 7:**
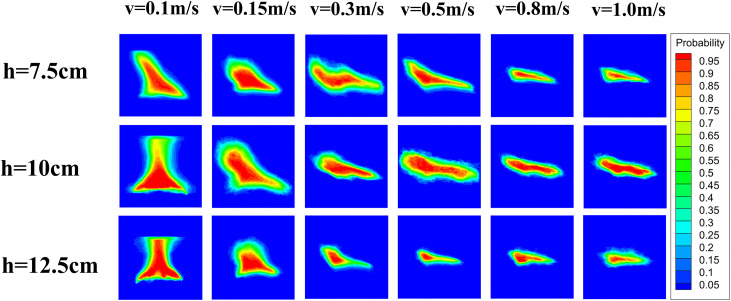
Flame probability contour under each operating condition.

As shown in Figs 5–7, flames generated by moving fire sources exhibit pronounced tilting behaviors compared to those produced by stationary sources. Under constant fire source height conditions, increasing the movement speed leads to greater flame tilt and lateral displacement, as well as a reduction in flame length and a progressive weakening of combustion intensity. The flame morphology transitions from a tongue-like structure to a more flattened shape. This phenomenon can be attributed to the interaction between the moving fire source and the surrounding airflow. The movement induces a disturbance in the ambient air, generating a piston effect that drives airflow in the direction opposite to the fire source’s motion. This airflow causes the flame to tilt backward and elongate into a tongue-like form. As the movement speed increases, the strength of the airflow disturbance and the associated piston wind intensify, resulting in a more pronounced flame tilt. Once the movement speed exceeds a critical threshold, the intensified airflow begins to suppress combustion, leading to a transformation of the flame shape from tongue-like to flattened.

The influence of movement speed on flame morphology varies with different fire source heights. The critical speed $v_{c}$, at which a significant reduction in flame height occurs, differs across height conditions: for h=7.5 cm, $v_{c}=0.8$ m/s; for h=10 cm, $v_{c}=1.0$ m/s; and for h=12.5 cm, $v_{c}$ decreases to 0.3 m/s. Similarly, the critical speed $v_{t}$, at which the flame tilt becomes markedly pronounced, also varies: for h=7.5 cm, $v_{t}=0.8$ m/s; for h=10 cm, $v_{t}=1.0$ m/s; and for h=12.5 cm, $v_{t}=0.5$ m/s. In addition, flame brightness decreases with increasing movement speed. This reduction is especially evident at fire source heights of 7.5 cm and 12.5 cm.

In contrast, at a fire source height of 10 cm, the flame showed the highest brightness and maintained a relatively stable structure with the maximum vertical extension, as quantified in Fig 7. The simultaneous increase in flame brightness and height indicates an enhancement in macroscopic combustion intensity. This behavior is attributed to a favorable aerodynamic condition. Raising the fire source to 10 cm increases the local blockage ratio, which promotes vortex entrainment and improves fuel–air mixing. At higher elevations (12.5 cm), stronger shear from the ventilation core tends to weaken the flame. In comparison, the 10 cm height provides a balance, where air entrainment is enhanced without strong shear suppression, resulting in a more fully developed flame structure.

**Fig 8:**
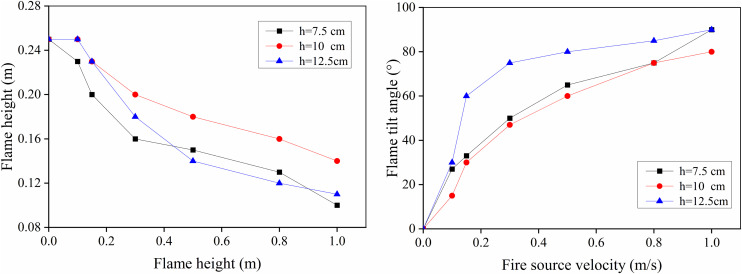
The flame height and inclination vary with the height of the fire source and the speed of movement. (a) Flame height changes (b) Changes in flame inclination.

To further examine the effects of fire source height and movement speed on flame geometry, a quantitative analysis of flame height and tilt angle was performed. The quantitative values were obtained by averaging measurements extracted from multiple consecutive instantaneous frames. This averaging method was used to reduce the influence of transient flame flickering and frame-to-frame variations. All values were calculated using the pixel-to-meter conversion determined during camera calibration. The results of this analysis are presented in Fig 8.

Fig 8 shows that, with constant fire source height, flame height decreased as movement speed increases, while the flame tilt angle increases. Fig 8(a) further indicates that as fire source height rises, flame height first increases and then decreases. When the fire source height increased from 7.5 cm to 10 cm, the flame height increased by 8.70%, 15.00%, 25.00%, 20.00%, 23.08%, and 40.00% under the moving speed conditions of 0.1 m/s, 0.15 m/s, 0.3 m/s, 0.5 m/s, 0.8 m/s, and 1.0 m/s, respectively. The core mechanism driving this phenomenon is that elevating the fire source (from 7.5 cm to 10 cm) enhances the interaction between the flame and the local flow field. This elevation exposes the combustion zone to a more active flow region and slightly increases the cross-sectional blockage ratio. These changes induce moderate airflow disturbance and vortex entrainment around the fuel pan, optimizing fuel-oxygen mixing efficiency and accelerating the fuel mass loss rate [13,15]. Consequently, combustion intensity is strengthened, promoting a corresponding rise in flame height. However, when the fire source height reaches 12.5 cm, the excessive blockage ratio significantly constricts the airflow passage, causing a sharp increase in local flow velocity. This triggers intense convective heat loss, the negative impact of which outweighs the benefits of enhanced mixing, ultimately weakening combustion and causing a sharp decline in flame height due to quenching effects.

As illustrated in Fig 8(b), the flame tilt angle initially decreases and then increases with the height of increasing fire source. The minimum tilt angle is observed at a height of 10 cm, followed by 7.5 cm, while the maximum occurs at 12.5 cm. This trend can be explained by the interplay between combustion stability and airflow disturbance. When the fire source height increases from 7.5 cm to 10 cm, the combustion becomes more stable and intense, as previously discussed. Under these conditions, the influence of fire source movement on the surrounding airflow is relatively limited, resulting in a reduced flame tilt angle. However, when the fire source height further increases to 12.5 cm, the airflow disturbance intensifies due to the increased blockage ratio. Consequently, the flame experiences stronger horizontal airflow, which amplifies the tilt angle. Furthermore, the increase in fire source height reduces the available space for upward air movement, leading to a decrease in oxygen supply and suppression of combustion. To compensate for the limited oxygen, the flame stretches laterally into the surrounding space, further increasing its tilt angle.

In summary, when the height of fire source remains constant, the flame characteristics are markedly affected by the movement speed. As the speed increases, the flame shape gradually transitions from a tongue-like form to a flattened profile, accompanied by an increase in tilt angle and a reduction in flame length. When the fire source height is below 10 cm, increasing the height enhances combustion by improving airflow mixing. However, once the fire source height exceeds 10 cm, further increases suppress combustion due to intensified airflow disturbance and reduced oxygen availability. Consequently, flame height exhibits a non-monotonic trend—increasing initially and then decreasing with increasing fire source height—while the flame tilt angle shows the opposite pattern, decreasing first and then increasing.

### 3.2. Analysis of influencing factors of flame height

The effective tunnel height *H* is defined as the vertical distance from the fire source to the tunnel ceiling. For varying fire source heights and movement speeds, two key influencing parameters are introduced: *H* and the speed *v*. Under moving fire source conditions, it is assumed that the flame height *L* is also related to the heat release rate *Q*, air density ρ, gravitational acceleration *g*, specific heat capacity at constant pressure *c*_*p*_, ambient temperature $T_{\text{0}}$ and pool fire source area *D*. Accordingly, the flame height *L* is considered to be a function of these variables, and the relationship can be expressed as shown in Eq. (2).


$$f\left(L,Q,H\text{,}v,\rho ,c_{\text{p}},T_{\text{0}}\text{,g,}D\right)=0$$ (2)

Using mass dimension M, time dimension T, length dimension L, and thermodynamic temperature Θ as the fundamental dimensions, the dimensional expressions of $L,\text{\textbackslash{}hspace\{0.17em}\}Q,\text{\textbackslash{}hspace\{0.17em}\}H,\text{\textbackslash{}hspace\{0.17em}\}v,\text{\textbackslash{}hspace\{0.17em}\}\rho ,\text{\textbackslash{}hspace\{0.17em}\}c_{\text{p}},\text{\textbackslash{}hspace\{0.17em}\}T_{\text{0}},\text{\textbackslash{}hspace\{0.17em}g,\;\}D$ can be represented as *L*, *ML*^*2*^*T*^*−3*^*, L, LT*^*-1*^*, ML*^*-2*^*, L*^*2*^*T*^*-2*^*Θ*^*-1*^, Θ, *LT*^*-2*^*, L*. By selecting these as the fundamental variables and applying the π-theorem [41], the unknown parameters *π*_*1*_ ~ π_*5*_ can be expressed as shown in Eq. (3).


$$\left\{ \begin{array}{c} @l@\pi_{\text{1}}=H^{a_{\text{1}}}v^{\beta_{\text{1}}}\rho_{\text{0}}^{\gamma_{\text{1}}}c_{p}^{\delta_{\text{1}}}L\mathrm{\backslash vspace}1.5mm\\ \pi_{\text{2}}=H^{a_{\text{2}}}v^{\beta_{\text{2}}}\rho_{\text{0}}^{\gamma_{\text{2}}}c_{p}^{\delta_{\text{2}}}Q\mathrm{\backslash vspace}1.5mm\\ \pi_{\text{3}}=H^{a_{\text{3}}}v^{\beta_{\text{3}}}\rho_{\text{0}}^{\gamma_{\text{3}}}c_{p}^{\delta_{\text{3}}}T_{\text{0}}\mathrm{\backslash vspace}1.5mm\\ \pi_{\text{4}}=H^{a_{\text{4}}}v^{\beta_{\text{4}}}\rho_{\text{0}}^{\gamma_{\text{4}}}c_{p}^{\delta_{\text{4}}}g\mathrm{\backslash vspace}1.5mm\\ \pi_{\text{5}}=H^{a_{\text{5}}}v^{\beta_{\text{5}}}\rho_{\text{0}}^{\gamma_{\text{5}}}c_{p}^{\delta_{\text{5}}}D \end{array}\right.$$ (3)

By performing dimensional analysis on Eq. (3), the expressions for *π*_*1*_ ~ π_*5*_ are obtained as shown in Eq. (4):


$$\left\{ \begin{array}{c} @l@\pi_{\text{1}}=H^{-1}L\\ \pi_{\text{2}}=H^{-1}v^{-3}\rho^{-1}Q\mathrm{\backslash vspace}0.5mm\\ \pi_{\text{3}}=v^{-2}c_{p}T_{\text{0}}\mathrm{\backslash vspace}1.0mm\\ \pi_{\text{4}}=Hv^{-2}g\\ \pi_{\text{5}}=H^{-1}D \end{array}\right.$$ (4)

By substituting Eq. (4) into Eq. (2), we obtain:


$$f\left(H^{-1}L_{\text{0}},H^{-1}v^{-3}\rho^{-1}Q,v^{-2}c_{p}T_{\text{0}},Hv^{-2}g,H^{-1}D\right)\;=0$$ (5)

Eq. (5) establishes that the relationship among the independent dimensionless groups equals zero, representing an implicit function $f\left(\pi_{\text{1}},\pi_{\text{2}},\ldots \right)=0$. According to the mathematical properties of implicit functions, the specific dimensionless group ($\pi_{\text{1}}=H^{-1}L$) can be expressed as an explicit function of the other governing parameters. Therefore, Eq. (5) implies the existence of the functional relationship shown in Eq. (6). To physically characterize the flow, the independent variables on the right-hand side are grouped [41] to form a composite dimensionless group ($\frac{QHg}{v^{\text{3}}\rho c_{p}T_{\text{0}}D}$), which physically represents the ratio of buoyancy flux to inertial flux, leading to Eq. (6),


$$\begin{array}{c} H^{-1}L=f\left(H^{-1}v^{-3}\rho^{-1}Q,v^{-2}c_{p}T_{\text{0}},Hv^{-2}g\cdot HD^{-1}\right)\\ \;\;\;=f\left(H^{-1}v^{-3}\rho^{-1}Q,v^{-2}c_{p}T_{\text{0}}\cdot H^{-2}v^{\text{2}}g^{-1}D\right)\\ \;\;\;=f\left(H^{-1}v^{-3}\rho^{-1}Q\cdot {c_{p}}^{-1}{T_{\text{0}}}^{-1}H^{\text{2}}gD^{-1}\right)=f\left(\frac{QHg}{v^{\text{3}}\rho c_{p}T_{\text{0}}D}\right) \end{array}$$ (6)

Thus, the relationship is expressed as shown in Eq. (7):


$$H^{-1}L=f\left(\frac{QgH}{v^{\text{3}}\rho c_{p}T_{\text{0}}D}\right)\;$$ (7)

**Fig 9:**
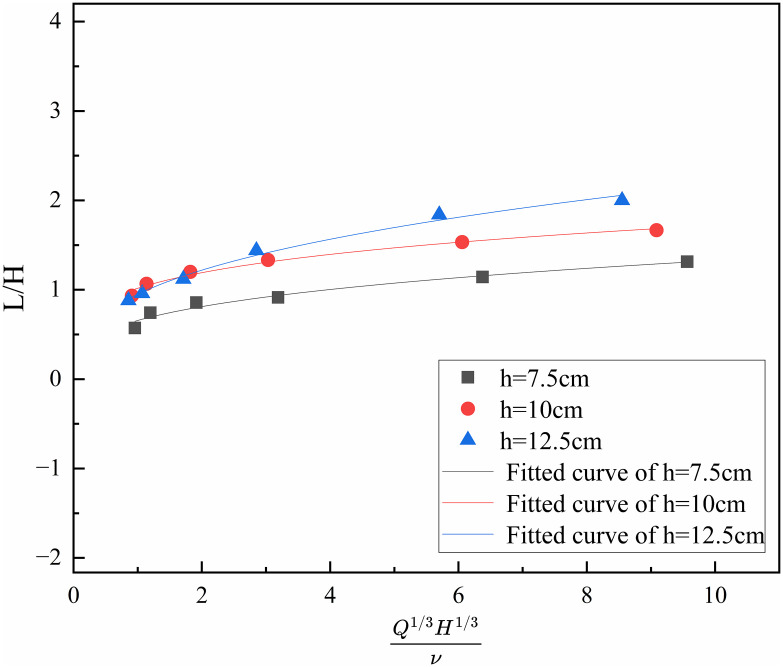
Fitting curves of the relationship between flame height, the effective height of the tunnel and ignition moving velocity at different heights.

Under standard atmospheric pressure and room temperature conditions, $\rho ,\text{\textbackslash{}hspace\{0.17em}\}c_{\text{p}},\text{\textbackslash{}hspace\{0.17em}\}T_{\text{0}},\text{\textbackslash{}hspace\{0.17em}g\}$ can be considered a constant. At this point, the ratio of *L* to *H*^*-1*^is related to $QH/v^{\text{3}}D$ and follows a certain functional relationship. During the experiment, with the oil pan size remaining constant (i.e., D is a constant), the ratio of flame height to effective tunnel height is related to L/H and $\frac{QH}{v^{\text{3}}}$ ($\frac{Q^{\frac{\text{1}}{\text{3}}}H^{\frac{\text{1}}{\text{3}}}}{v}$). By processing the relevant data, the corresponding relationship between L/H and $\frac{Q^{\frac{\text{1}}{\text{3}}}H^{\frac{\text{1}}{\text{3}}}}{v}$ was obtained. The fitted curves of flame height versus *H* and *v* at different heights are shown in Fig 9.

Under different fire source height conditions, the relationship between flame height, effective tunnel height, and *v* follows the form of $y=ax^{b}$. The corresponding relationships between flame height, effective tunnel height (the distance from the fire source to the tunnel ceiling), and *v* under different fire source height conditions are shown in Eqs. (8) ~ (10)


$$\frac{L}{H}=0.66{\left(\frac{Q^{\frac{\text{1}}{\text{3}}}H^{\frac{\text{1}}{\text{3}}}}{v}\right)}^{0.3}$$ (8)

$$\frac{L}{H}=0.92{\left(\frac{Q^{\frac{\text{1}}{\text{3}}}H^{\frac{\text{1}}{\text{3}}}}{v}\right)}^{0.3}$$ (9)

$$\frac{L}{H}=1.02{\left(\frac{Q^{\frac{\text{1}}{\text{3}}}H^{\frac{\text{1}}{\text{3}}}}{v}\right)}^{0.3}$$ (10)

**Fig 10:**
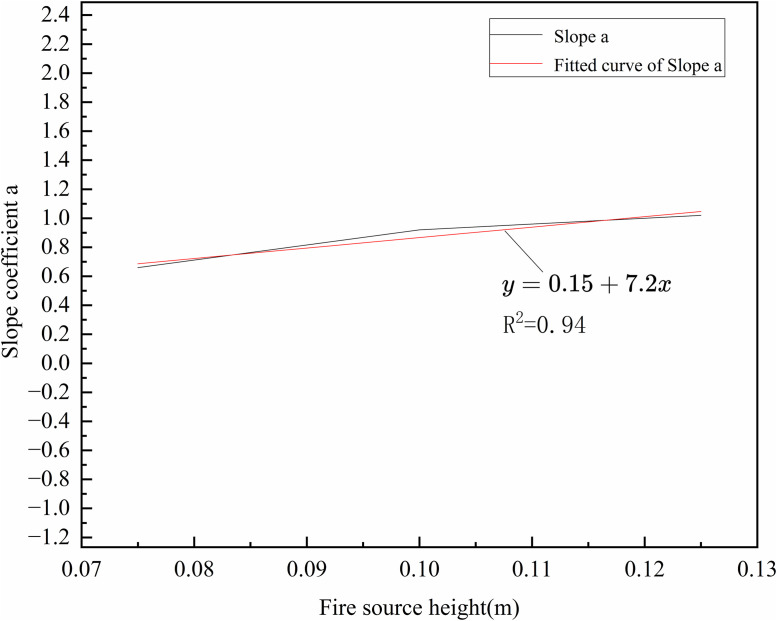
Fitting results of the slope a of the modified formula at different heights.

As shown in Eqs. (8) ~ (10), the *a* corresponding to fire source heights of 7.5 cm, 10 cm, and 12.5 cm are 0.66, 0.92, and 1.02, respectively. It is evident that the slope *a* increases with increasing fire source height, indicating a stronger dependence of flame height on *v* at greater elevations. The fitted values of the slope *a* under different fire source height conditions are illustrated in Fig 10.

Therefore, the relationship between flame height, effective tunnel height, and *v* can be expressed as:


$$\frac{L}{H}=\left(0.15+7.2\text{h}\right){\left(\frac{Q^{\frac{\text{1}}{\text{3}}}H^{\frac{\text{1}}{\text{3}}}}{v}\right)}^{0.3}$$ (11)

The results indicate that the ratio of flame height to effective tunnel height within the tunnel is generally directly proportional to the fire source height and inversely proportional to the *v*. Compared to previous studies [36], the observed trend is consistent: the ratio increases with increasing fire source height and decreases as movement speed increases. This further validates the general applicability of the identified relationship under different experimental conditions.

The errors between the ratio of flame height to effective tunnel height predicted by the statistical formula and the actual data are presented in Table 3.

**Table 3:** Statistics of Prediction Errors for the Ratio of Flame Height to Effective Tunnel Height.

	h = 7.5 cm		h = 10 cm		h = 12.5 cm	
	Error (cm)	Ratio (%)	Error (cm)	Ratio (%)	Error (cm)	Ratio (%)
**v = 0.1 m/s**	0.01	1.13	−0.12	−7.02	0.06	2.91
**v = 0.15 m/s**	−0.01	−0.68	−0.05	−3.00	0.12	6.56
**v = 0.3 m/s**	−0.02	−2.22	0.05	3.79	0.04	3.02
**v = 0.5 m/s**	0.06	6.46	0.10	8.29	−0.08	−6.97
**v = 0.8 m/s**	0.05	6.26	0.11	10.39	−0.08	−8.39
**v = 1.0 m/s**	−0.08	−13.97	0.04	4.22	−0.09	−10.59

**Fig 11:**
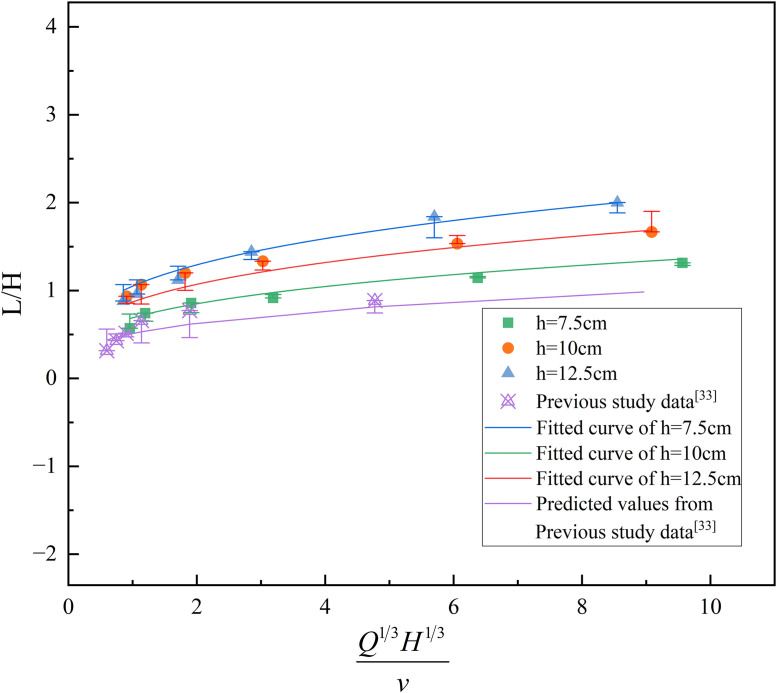
Comparison on results of this model and previous research.

As shown in Fig 11 and Table 3, the experimental data are in good agreement with the fitting curve, and the errors under different fire source heights are all within a small range. The errors between the prediction formula and experimental results mainly originate from three aspects: first, the inherent errors of measuring instruments, such as the precision deviation of thermocouples, the speed control error of variable-speed motors, and the edge recognition error possibly caused by camera resolution when measuring flame height via the combination of a laser emitter and a camera; second, the human errors in experimental operation and measurement processes; third, the fitting errors generated during data fitting.

To reduce the impact of the above errors on the results, this study repeated each working condition 4 times to minimize random errors: by calculating the average value of the 4 repeated experimental data and verifying the standard deviation range, the accidental errors that might exist in a single experiment were eliminated, making the final results closer to the true values. The repeated experimental results showed that the intra-group standard deviations of key parameters were all smaller than the inherent errors of the measuring instruments, indicating that random errors had been effectively controlled.

Meanwhile, as seen in Table 3, the errors under most working conditions are within ±10%, which indicates that the predicted results of Eq. (11) are in good consistency with the experimental measured values, and the established prediction model has certain accuracy, which can predict the ratio of flame height to effective tunnel height in tunnel fires caused by moving fire sources with different heights to a certain extent. In addition, the predicted results of this model are close to the data from previous studies; although there is a certain deviation between the predicted values and measured values due to differences in experimental conditions (e.g., scale ratio, fuel type), the deviation range is small, which further verifies the ability of the model to predict the ratio of flame height to effective tunnel height in tunnel fires with moving fire sources of different heights.

### 3.3. Analysis of smoke propagation process

**Fig 12:**
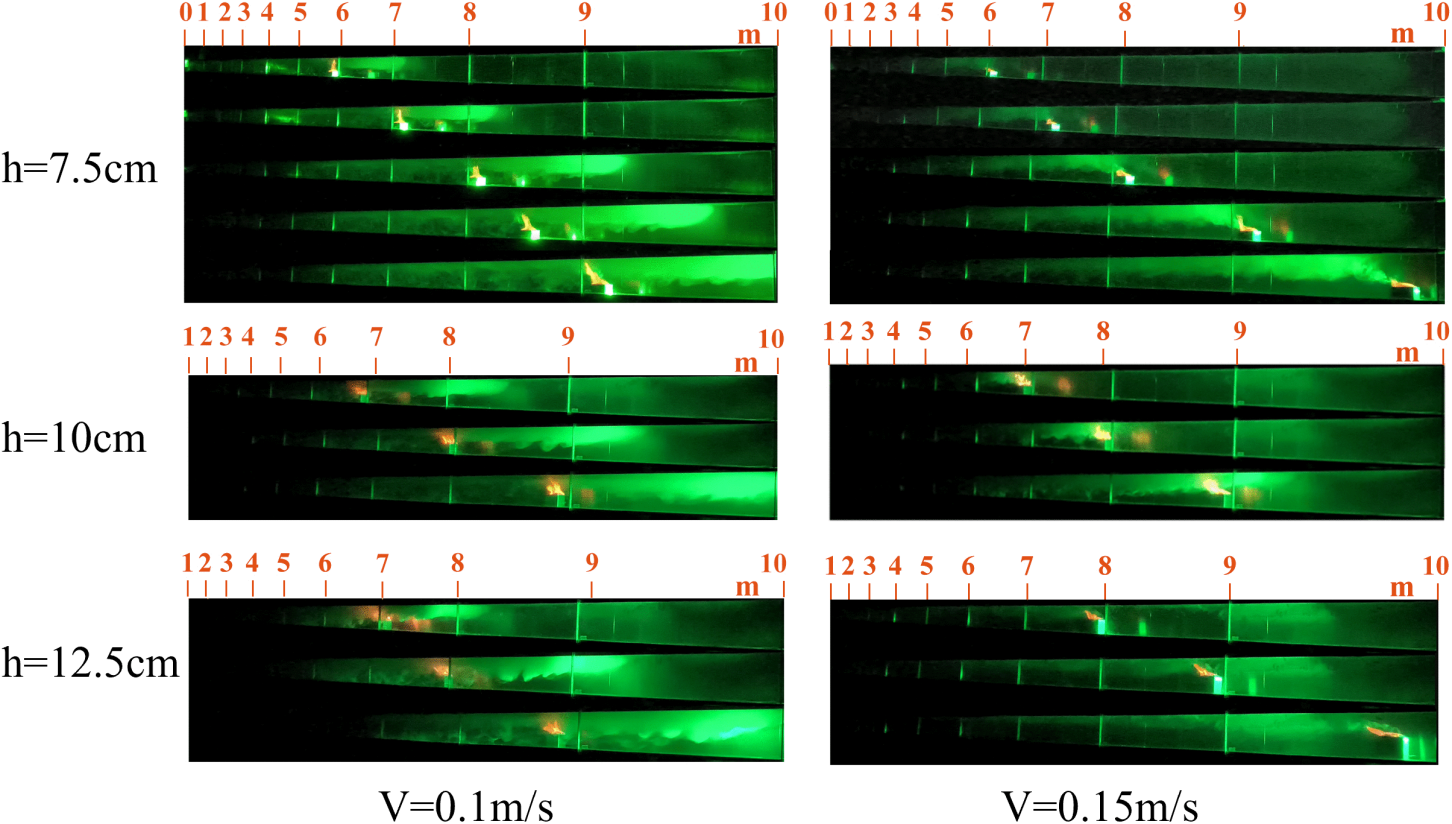
Smoke spread When the speed is 0.1 m/s and 0.15 m/s.

**Fig 13:**
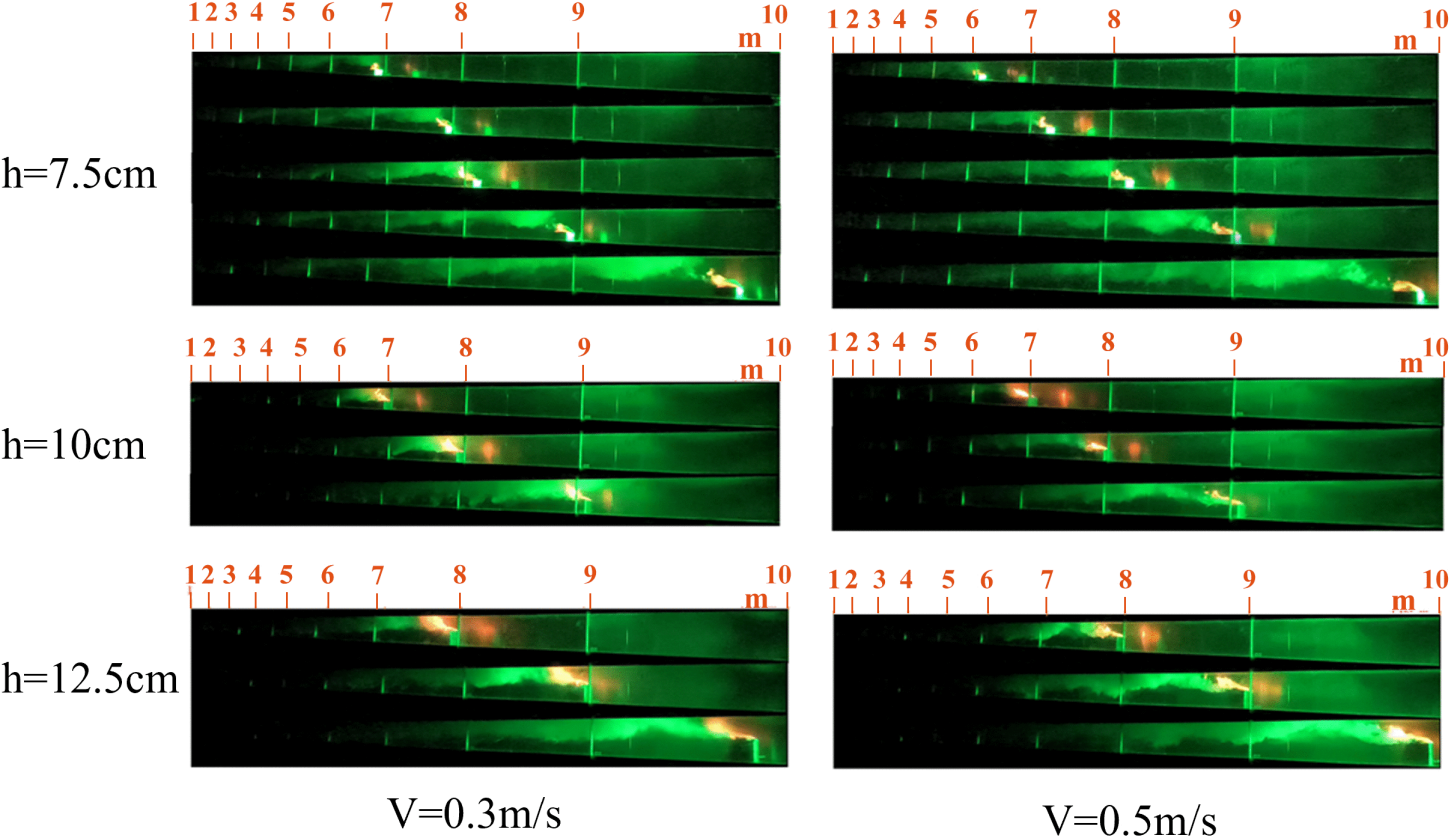
Smoke spread When the speed is 0.3 m/s and 0.5 m/s.

**Fig 14:**
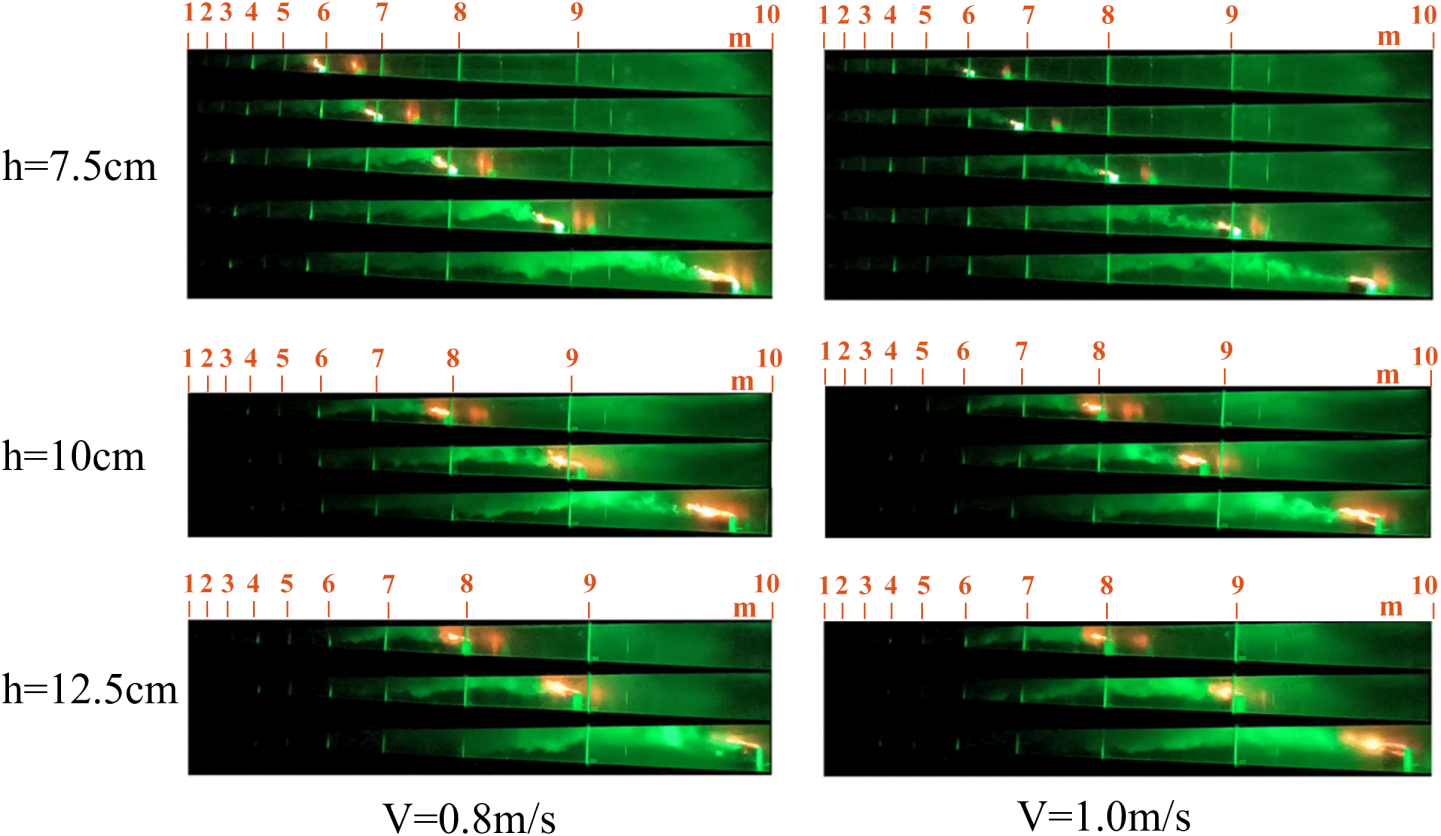
Smoke spread When the speed is 0.8 m/s and 1.0 m/s.

The smoke propagation for speeds of 0.1 m/s, 0.15 m/s, 0.3 m/s, 0.5 m/s, 0.8 m/s, and 1.0 m/s, with fire source heights of 7.5 cm, 10 cm, and 12.5 cm, is shown in Figs 12–14.

**Fig 15:**
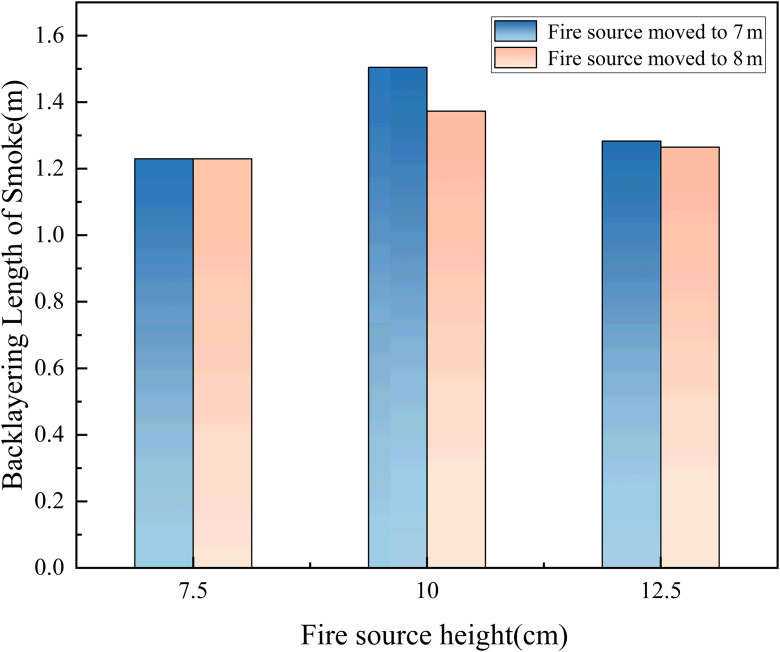
Smoke backflow distance when *V* =  0.1 m/s.

Figs 12–14 show that fire source movement causes a pronounced tilt in the flame plume. Previous studies indicate that at low speeds, smoke in confined tunnels propagates both upstream and downstream, forming backflow. However, when the speed exceeds a certain threshold, backflow disappears [23]. The smoke backflow length is defined as the maximum longitudinal distance of reverse smoke spread upstream of the fire source, specifically referring to the horizontal distance from the center of the fire source to the upstream smoke front (i.e., the smoke boundary identified via laser visualization technology). As shown in Fig 12, smoke backflow occurs when the fire source moves at 0.1 m/s. The backflow distances at fire source heights of 7 m and 8 m are calculated and presented in Fig 15.

As illustrated in Fig 15, when the fire source moves at a speed of 0.1 m/s, the smoke backflow distance initially increases and then decreases with increasing fire source height. The maximum backflow distance is observed at a fire source height of 10 cm, followed by 12.5 cm, and is lowest at 7.5 cm. This trend can be explained as follows: increasing the fire source height reduces the vertical distance that smoke must travel to reach the tunnel ceiling, allowing the ceiling-accumulated smoke to flow backward more rapidly toward the leading edge of the fire plume. However, when the fire source height exceeds 10 cm, the reduced gap between the flame and the ceiling limits oxygen availability, thereby lowering combustion efficiency. Simultaneously, the increased flame tilt causes the flame plume to impact the ceiling further downstream, resulting in a shorter smoke backflow distance.

To evaluate upstream smoke propagation, the measured backflow distance was compared with the theoretical prediction for a stationary fire based on the model proposed by Li et al. (2010) [42]. Under an equivalent low ventilation condition (where the fire source movement speed is directly treated as the ventilation velocity), the stationary model predicts a much longer back-layering length. This indicates that, under weak airflow inertia, smoke can spread significantly upstream. In contrast, the backflow distance measured in the present experiments is much shorter. This difference confirms that the piston effect induced by the moving vehicle generates a strong opposing dynamic pressure, which effectively suppresses upstream smoke propagation compared with the stationary fire scenario.

**Fig 16:**
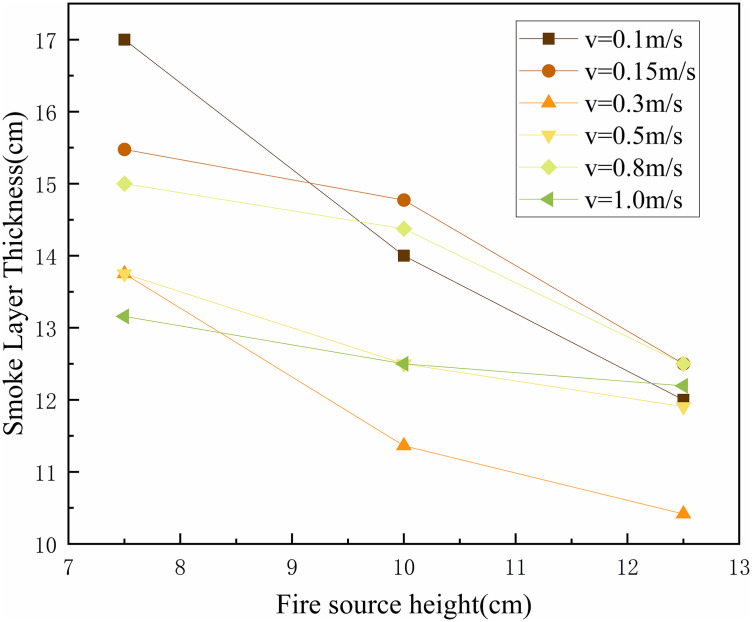
The thickness of the smoke when the ignition source is moved to 8 m.

In tunnel fire scenarios, the thickness of smoke layer is a critical parameter that directly influences evacuation safety, fire fighting effectiveness, and the structural integrity of the tunnel. Therefore, understanding the variation in smoke layer thickness is essential for developing effective emergency response strategies and optimizing tunnel fire safety design. The smoke layer thickness is defined as the vertical distance between the tunnel ceiling and the lower boundary of the high-temperature smoke layer. The lower boundary of the smoke layer was identified using the laser sheet visualization technique. A laser emitter installed at the tunnel end generated a longitudinal light sheet that illuminated the smoke particles. The clear interface between the smoke layer and the ambient air, enhanced by light scattering (Tyndall effect), was captured by a high-definition camera. The smoke layer thickness was then determined by analyzing the pixel coordinates of this illuminated boundary in the recorded images. In this study, the smoke layer thickness at the 8 m position during fire source movement was measured and statistically analyzed. The results under various conditions are presented in Fig 16.

Fig 16 shows that, under constant speed conditions, the smoke layer thickness decreased as the fire source height increases. At a speed of 0.1 m/s, when the fire source reaches 8 m, the smoke layer thickness is approximately 0.17 m for a height of 7.5 cm, 0.14 m at 10 cm, and 0.12 m at 12.5 cm. This trend can be attributed to changes in plume dynamics as the fire source height increases. At lower heights, the plume travels a longer vertical distance before reaching the ceiling, reducing its upward momentum and resulting in more vertical diffusion, which leads to a thicker smoke layer. As the fire source height increases, the ascent path shortens, and the plume reaches the ceiling with greater velocity. This increases the horizontal spread of smoke along the ceiling, enhancing lateral dispersion and reducing vertical accumulation, which thins the smoke layer. Additionally, as the gap between the fire source and the ceiling decreases, the smoke cooling time is reduced, leading to higher temperatures near the ceiling. The increased thermal buoyancy prevents the smoke from descending, causing it to accumulate in the upper region of the tunnel, further thinning the smoke layer.

### 3.4. Temperature analysis of the tunnel roof

**Fig 17:**
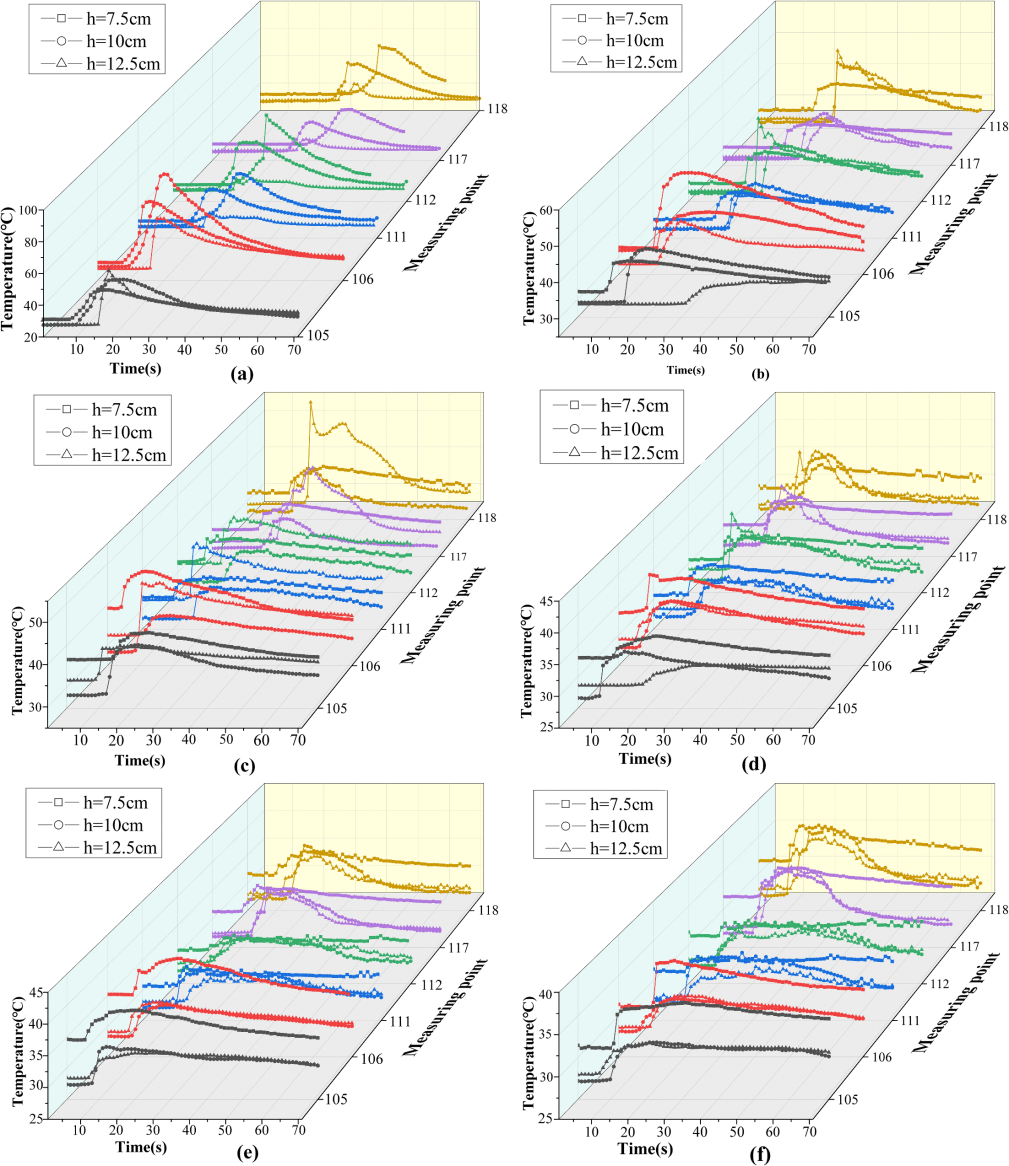
Temperature variation diagram of different measurement points. (a) v=0.1 m/s; (b) v=0.15 m/s; (c) v=0.3 m/s; (d) v=0.5 m/s; (e) v=0.8 m/s; (f) v=1.0 m/s.

Real-time temperature variations within the model were monitored using K-type thermocouples. Temperature data were collected at measurement points 105, 111, and 117, which are positioned near the tunnel sidewalls, as well as at points 106, 112, and 118, located along the ceiling centerline. The recorded temperature profiles under different experimental conditions are presented in Fig 17.

Fig 17 shows that the speed markedly affects the timing between the onset of ceiling temperature rise and the moment the fire source reaches the measurement point. At lower speeds, the ceiling temperature rises before the fire source arrives directly beneath the corresponding point. In contrast, as the movement speed increases beyond a certain threshold, the ceiling temperature rise is delayed relative to the fire source’s arrival. For example, at a movement speed of 0.1 m/s, the influence of smoke backflow causes the temperature at the ceiling to rise approximately 15 s before the fire source reaches the location directly below the measurement point. However, when the movement speed increases to 0.5 m/s, the flame plume exhibits significant tilt, and the plume impact zone shifts downstream. As a result, the temperature rise is delayed by approximately 4 s relative to the fire source’s arrival at the measurement location. This phenomenon is primarily attributed to changes in the dominant heat transfer mechanism. At low fire source speeds, upstream smoke backflow transports thermal energy ahead of the flame front, resulting in early temperature rise at ceiling-level measurement points. As the speed increases, the intensified tilt of the flame plume displaces the primary heat transfer region further downstream. Consequently, thermal energy reaches the ceiling measurement point later, leading to a noticeable delay in temperature response.

As shown in Fig 17, speed has a significant influence on the peak temperature within the tunnel. At a low movement speed of 0.1 m/s, the highest peak temperature is observed, reaching 89.4°C. In contrast, when the movement speed increases to 1.0 m/s, the peak temperature decreases substantially, reaching only 37°C. This reduction is primarily due to the shortened residence time of the flame within the tunnel at higher speeds, which reduces the duration of heat release. Additionally, increased movement speed enhances airflow disturbance, thereby accelerating convective heat exchange between hot smoke and the surrounding cold air, further contributing to the temperature drop. Moreover, as speed increases, the influence of fire source height on ceiling temperature becomes progressively less significant. At a speed of 0.1 m/s, the maximum temperature difference among the three fire source heights reaches approximately 20°C (Fig 17. However, at 1.0 m/s, this difference is reduced to around 2°C. This behavior can be attributed to the dominance of different mechanisms at varying speeds: when the movement speed is low (v ≤ 0.15 m/s), flame combustion is strongly affected by fire source height, resulting in large temperature disparities. In contrast, at higher speeds (v ≥ 0.15 m/s), the combustion and heat distribution are more strongly governed by the fire source’s movement dynamics, leading to smaller temperature differences across different heights.

Furthermore, the measured ceiling temperatures were compared with the stationary tunnel fire model proposed by Li et al. (2011) [3]. The stationary model predicts peak ceiling temperatures that are generally higher than those measured in the present experiments. This clear difference highlights the “scanning effect” of a moving fire source. Unlike stationary fires, where the plume continuously heats a fixed ceiling region, a moving fire source has a limited residence time at any given location. As a result, the ceiling does not accumulate enough heat to reach the thermal equilibrium assumed in stationary correlations.

**Fig 18:**
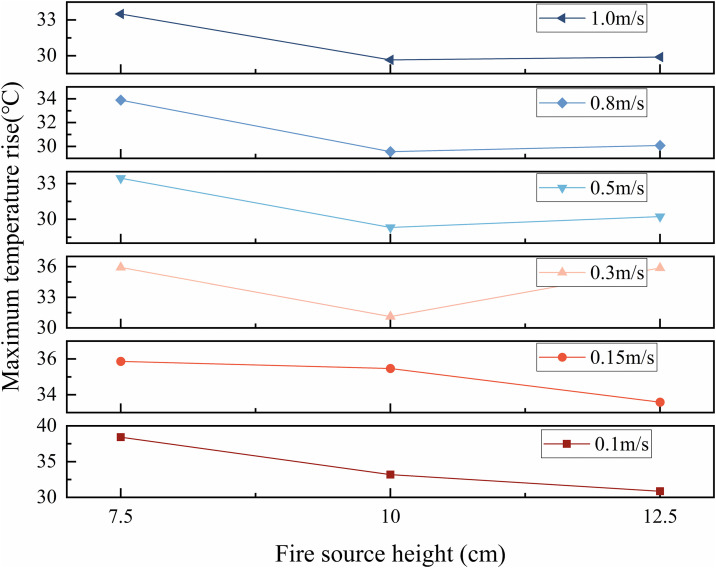
The average temperature of the measurement point under each working condition at 60 s.

To further examine the influence of fire source speed and height on ceiling temperature, temperature readings at each measurement point were recorded at 60 s, when thermal conditions had stabilized. The average ceiling temperatures under each experimental condition were summarized in Fig 18.

As shown in Fig 18, when the tunnel ceiling temperature reaches a quasi-steady state at 60 s, the average temperature under each operating condition varies with the speed. The results can be categorized into three distinct regimes: low speed (v ≤ 0.15 m/s), medium speed (0.15 m/s < v ≤ 0.5 m/s), and high speed (v > 0.5 m/s).

In the low-speed regime, flame behavior is markedly influenced by fire source height. As the height increases, the enhanced piston wind effect intensifies convective mixing between hot smoke and surrounding cold air, leading to a reduction in ceiling temperature.

**Fig 19:**
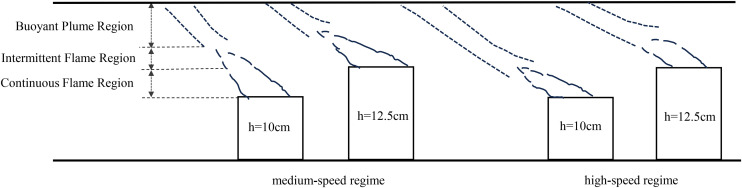
Fire plume partition in the inclined state.

In the medium-speed regime, flame behavior is primarily controlled by the speed. The average ceiling temperature shows a non-monotonic trend with increasing fire source height, initially decreasing and then increasing. At a height of 7.5 cm, the airflow disturbance is relatively weak, resulting in reduced thermal exchange and a higher tunnel temperature compared to the 10 cm and 12.5 cm heights. In this speed range, flame combustion at 10 cm and 12.5 cm remains stable. Additionally, because the continuous flame zone temperature is typically higher [43], and as shown in Fig 19, the fire plume at 12.5 cm impacts the ceiling closer to this zone, the temperature at 12.5 cm exceeds that at 10 cm.

In the high-speed regime, the trend is similar to that observed under medium-speed conditions, with combustion dynamics primarily affected by movement speed. The tunnel temperature remains highest at a fire source height of 7.5 cm. However, unlike the medium-speed regime, the temperature difference between fire source heights of 10 cm and 12.5 cm becomes negligible. Although the plume at 12.5 cm still impacts the ceiling near the continuous flame zone and reaches high local temperatures (Fig 19), the overall combustion intensity at this height weakens markedly at high speed. In contrast, combustion remains relatively robust at 10 cm, sustaining higher average temperatures. As a result, the temperature gap between these two heights narrows.

### 3.5. Limitations and perspectives

Despite the insights provided by the present study, several limitations should be acknowledged. First, the experiments were conducted using a reduced-scale tunnel model designed according to the Froude similarity criterion. While this approach is widely adopted to capture buoyancy–inertia-dominated flow behavior in tunnel fires, the present study is not intended to provide direct quantitative predictions for full-scale tunnel fire scenarios. Instead, it should be regarded as an exploratory investigation into the macroscopic influence of fire source movement speed and elevation on tunnel fire behavior, with emphasis on physical trends and dominant flow–flame interaction mechanisms. Extrapolation of the present findings to real tunnel fires therefore requires further efforts, such as full-scale experimental validation, refined similarity considerations, or combined numerical–experimental studies.

Second, the fire source configuration was simplified to a single, steadily moving pool fire with a fixed heat release rate and a limited range of elevations. In real tunnel fires, vehicle combustion may involve unsteady heat release, complex geometries, fuel heterogeneity, and interactions with ventilation systems and surrounding traffic. Although such simplifications are necessary to isolate the coupled effects of fire source height and movement speed, they inevitably constrain the generality of the conclusions. Future studies incorporating a wider range of fire source powers, elevation distributions, and ventilation conditions are therefore warranted.

Finally, compared with existing studies that mainly focus on either stationary elevated fire sources or moving fire sources at fixed heights, the present work specifically addresses the coupled effects of fire source height and movement speed, which remain insufficiently explored. While this coupling-oriented approach enhances the understanding of tunnel fire dynamics under moving fire scenarios, further systematic investigations are still required to bridge the gap between simplified experimental models and complex real-world tunnel fire conditions.

## 4. Conclusions

In this study, a previously established small-scale experimental platform, designed in accordance with Froude number similarity, was utilized to investigate the influence of simultaneous variations in fire source height and movement velocity on tunnel fire dynamics. Based on the experimental findings, the main conclusions are summarized as follows:

(1) As the fire source height increases, airflow disturbance becomes more intense, and the critical velocity at which flame weakening occurs is first delayed and then advanced. At a fixed fire source height, increasing movement speed results in a reduction in flame height and a significant increase in flame tilt angle and lateral deflection. Conversely, at a fixed movement speed, the flame tilt angle first decreases and then increases with increasing fire source height, while flame height shows the opposite trend—initially increasing and then decreasing. Furthermore, the ratio of flame height *L* to effective tunnel height *H* exhibits a functional relationship with $QH/v^{\text{3}}$.
(2) At low movement speeds, smoke backflow is observed, and the backflow distance initially increases and then decreases with increasing fire source height. A greater fire source height reduces the vertical entrainment path of the plume, thereby hindering the rapid cooling of high-temperature smoke. The strong thermal buoyancy of the hot gases limits their downward dispersion, leading to greater accumulation near the tunnel ceiling. As a result, the smoke layer becomes progressively thinner with increasing fire source height under identical movement speed conditions.
(3) At low movement speeds, tunnel temperature is primarily influenced by fire source height, whereas at higher speeds, the effect of movement speed becomes dominant. When the movement speed is low, the tunnel ceiling temperature tends to decrease with increasing fire source height. However, at higher speeds, the temperature exhibits a non-monotonic trend—first decreasing and then increasing. Notably, when the movement speed v ≥ 0.3 m/s, temperature fluctuations become more pronounced and intensify as the movement speed increases.

### 4.1. Appendix for uncertainty analysis

Uncertainty analysis was conducted in accordance with the method reported by Moffat [44]. Within the framework of this method, the total uncertainty can be determined via the root sum of squares (RSS) of all error components, with its calculation formula expressed as: $X_{total}=\sqrt{X_{\text{1}}^{\text{2}}+X_{\text{2}}^{\text{2}}+.......+X_{n}^{\text{2}}}$, where *n* denotes the total number of error sources. The relevant errors in this study have been calculated, statistically analyzed, and summarized in Table 4.

**Table 4:** Maximum uncertainty of parameters.

Parameters	Sources of uncertainty	Uncertainty value	Final uncertainty value
Q	Instrument precision error of electronic balance Assumption error of fuel combustion efficiency	±0.5% ±5.0%	±5.0%
L	Flame edge recognition error of camera-laser system Image pixel deviation error of camera resolution Datum error caused by fire source height measurement deviation	±5.0% ±3.0% ±2.0%	±6.2%
v	Speed control precision error of speed-regulating motor Signal error of speed measuring instrument (encoder) Disturbance of slight air flow in experimental environment on fire source movement	±2.0% ±1.0% ±1.0%	±2.4%
H	Measurement error of total tunnel model height Measurement deviation of fire source height Vertical offset error of fire source installation	±1.0% ±2.0% ±1.0%	±2.4%
L/H	Uncertainty transfer of heat release rate (Q) Uncertainty transfer of flame height (L) Direct measurement error of fire source velocity (v) Uncertainty transfer of effective tunnel height (H) Fitting error of L/H ratio	±5.0% ±6.2% ±2.4% ±2.4% ±10.0%	±13.2%

Analysis of Table 4 shows that the final uncertainty value of L/H is 13.2%, and this value is consistent with the actual error recorded in Table 3.

## Supporting information

S1 File Data. Numerical data used to generate the quantitative figures in the manuscript, including data for flame characteristics and smoke behavior.
(XLSX)

S2 File Temperature. Data for temperature analysis.
(XLSX)

S3 File Flame. Binary flame images used for the construction and analysis of flame probability contour maps.
(ZIP)
