# Supplementary figures and images for: Experimental investigation of tunnel fire spread under moving fire source conditions (part 2 of 2)
Source: PLoS One. 2026 Feb 24;21(2):e0336712. doi: 10.1371/journal.pone.0336712 (PMC12931806; doi:10.1371/journal.pone.0336712)

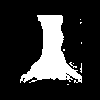

Supplement: S3 File — Binary flame images used for the construction and analysis of flame probability contour maps. (ZIP) [file pone.0336712.s003.zip › 10cm0.1/10_0.1101_cropped_cropped_cropped_adjusted.png]

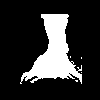

Supplement: S3 File — Binary flame images used for the construction and analysis of flame probability contour maps. (ZIP) [file pone.0336712.s003.zip › 10cm0.1/10_0.1102_cropped_cropped_cropped_adjusted.png]

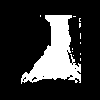

Supplement: S3 File — Binary flame images used for the construction and analysis of flame probability contour maps. (ZIP) [file pone.0336712.s003.zip › 10cm0.1/10_0.1103_cropped_cropped_cropped_adjusted.png]

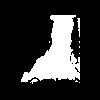

Supplement: S3 File — Binary flame images used for the construction and analysis of flame probability contour maps. (ZIP) [file pone.0336712.s003.zip › 10cm0.1/10_0.1104_cropped_cropped_cropped_adjusted.png]

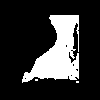

Supplement: S3 File — Binary flame images used for the construction and analysis of flame probability contour maps. (ZIP) [file pone.0336712.s003.zip › 10cm0.1/10_0.1105_cropped_cropped_cropped_adjusted.png]

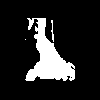

Supplement: S3 File — Binary flame images used for the construction and analysis of flame probability contour maps. (ZIP) [file pone.0336712.s003.zip › 10cm0.1/10_0.1113_cropped_cropped_adjusted.png]

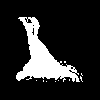

Supplement: S3 File — Binary flame images used for the construction and analysis of flame probability contour maps. (ZIP) [file pone.0336712.s003.zip › 10cm0.1/10_0.1114_cropped_cropped_adjusted.png]

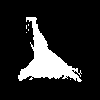

Supplement: S3 File — Binary flame images used for the construction and analysis of flame probability contour maps. (ZIP) [file pone.0336712.s003.zip › 10cm0.1/10_0.1115_cropped_cropped_adjusted.png]

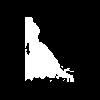

Supplement: S3 File — Binary flame images used for the construction and analysis of flame probability contour maps. (ZIP) [file pone.0336712.s003.zip › 10cm0.1/10_0.1116_cropped_cropped_adjusted.png]

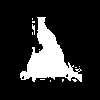

Supplement: S3 File — Binary flame images used for the construction and analysis of flame probability contour maps. (ZIP) [file pone.0336712.s003.zip › 10cm0.1/10_0.1117_cropped_cropped_adjusted.png]

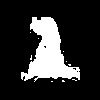

Supplement: S3 File — Binary flame images used for the construction and analysis of flame probability contour maps. (ZIP) [file pone.0336712.s003.zip › 10cm0.1/10_0.1118_cropped_cropped_adjusted.png]

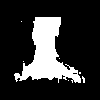

Supplement: S3 File — Binary flame images used for the construction and analysis of flame probability contour maps. (ZIP) [file pone.0336712.s003.zip › 10cm0.1/10_0.1119_cropped_cropped_adjusted.png]

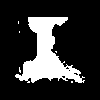

Supplement: S3 File — Binary flame images used for the construction and analysis of flame probability contour maps. (ZIP) [file pone.0336712.s003.zip › 10cm0.1/10_0.1120_cropped_cropped_adjusted.png]

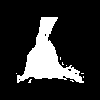

Supplement: S3 File — Binary flame images used for the construction and analysis of flame probability contour maps. (ZIP) [file pone.0336712.s003.zip › 10cm0.1/10_0.1121_cropped_cropped_adjusted.png]

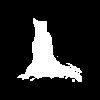

Supplement: S3 File — Binary flame images used for the construction and analysis of flame probability contour maps. (ZIP) [file pone.0336712.s003.zip › 10cm0.1/10_0.1122_cropped_cropped_adjusted.png]

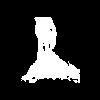

Supplement: S3 File — Binary flame images used for the construction and analysis of flame probability contour maps. (ZIP) [file pone.0336712.s003.zip › 10cm0.1/10_0.1123_cropped_cropped_adjusted.png]

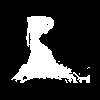

Supplement: S3 File — Binary flame images used for the construction and analysis of flame probability contour maps. (ZIP) [file pone.0336712.s003.zip › 10cm0.1/10_0.1124_cropped_cropped_adjusted.png]

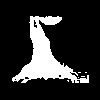

Supplement: S3 File — Binary flame images used for the construction and analysis of flame probability contour maps. (ZIP) [file pone.0336712.s003.zip › 10cm0.1/10_0.1125_cropped_cropped_adjusted.png]

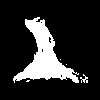

Supplement: S3 File — Binary flame images used for the construction and analysis of flame probability contour maps. (ZIP) [file pone.0336712.s003.zip › 10cm0.1/10_0.1126_cropped_cropped_adjusted.png]

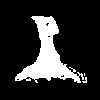

Supplement: S3 File — Binary flame images used for the construction and analysis of flame probability contour maps. (ZIP) [file pone.0336712.s003.zip › 10cm0.1/10_0.1127_cropped_cropped_adjusted.png]

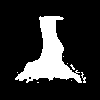

Supplement: S3 File — Binary flame images used for the construction and analysis of flame probability contour maps. (ZIP) [file pone.0336712.s003.zip › 10cm0.1/10_0.1128_cropped_cropped_adjusted.png]

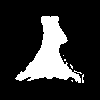

Supplement: S3 File — Binary flame images used for the construction and analysis of flame probability contour maps. (ZIP) [file pone.0336712.s003.zip › 10cm0.1/10_0.1129_cropped_cropped_adjusted.png]

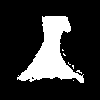

Supplement: S3 File — Binary flame images used for the construction and analysis of flame probability contour maps. (ZIP) [file pone.0336712.s003.zip › 10cm0.1/10_0.1130_cropped_cropped_adjusted.png]

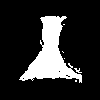

Supplement: S3 File — Binary flame images used for the construction and analysis of flame probability contour maps. (ZIP) [file pone.0336712.s003.zip › 10cm0.1/10_0.1131_cropped_cropped_adjusted.png]

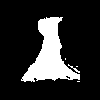

Supplement: S3 File — Binary flame images used for the construction and analysis of flame probability contour maps. (ZIP) [file pone.0336712.s003.zip › 10cm0.1/10_0.1132_cropped_cropped_adjusted.png]

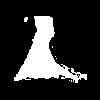

Supplement: S3 File — Binary flame images used for the construction and analysis of flame probability contour maps. (ZIP) [file pone.0336712.s003.zip › 10cm0.1/10_0.1133_cropped_cropped_adjusted.png]

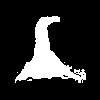

Supplement: S3 File — Binary flame images used for the construction and analysis of flame probability contour maps. (ZIP) [file pone.0336712.s003.zip › 10cm0.1/10_0.1134_cropped_cropped_adjusted.png]

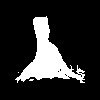

Supplement: S3 File — Binary flame images used for the construction and analysis of flame probability contour maps. (ZIP) [file pone.0336712.s003.zip › 10cm0.1/10_0.1135_cropped_cropped_adjusted.png]

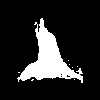

Supplement: S3 File — Binary flame images used for the construction and analysis of flame probability contour maps. (ZIP) [file pone.0336712.s003.zip › 10cm0.1/10_0.1136_cropped_cropped_adjusted.png]

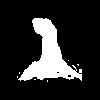

Supplement: S3 File — Binary flame images used for the construction and analysis of flame probability contour maps. (ZIP) [file pone.0336712.s003.zip › 10cm0.1/10_0.1137_cropped_cropped_adjusted.png]

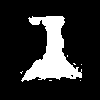

Supplement: S3 File — Binary flame images used for the construction and analysis of flame probability contour maps. (ZIP) [file pone.0336712.s003.zip › 10cm0.1/10_0.1138_cropped_cropped_adjusted.png]

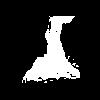

Supplement: S3 File — Binary flame images used for the construction and analysis of flame probability contour maps. (ZIP) [file pone.0336712.s003.zip › 10cm0.1/10_0.1139_cropped_cropped_adjusted.png]

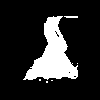

Supplement: S3 File — Binary flame images used for the construction and analysis of flame probability contour maps. (ZIP) [file pone.0336712.s003.zip › 10cm0.1/10_0.1140_cropped_cropped_adjusted.png]

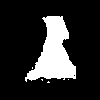

Supplement: S3 File — Binary flame images used for the construction and analysis of flame probability contour maps. (ZIP) [file pone.0336712.s003.zip › 10cm0.1/10_0.1141_cropped_cropped_adjusted.png]

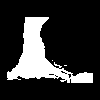

Supplement: S3 File — Binary flame images used for the construction and analysis of flame probability contour maps. (ZIP) [file pone.0336712.s003.zip › 10cm0.1/10_0.1142_cropped_cropped_adjusted.png]

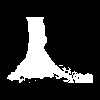

Supplement: S3 File — Binary flame images used for the construction and analysis of flame probability contour maps. (ZIP) [file pone.0336712.s003.zip › 10cm0.1/10_0.1143_cropped_cropped_adjusted.png]

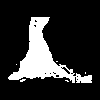

Supplement: S3 File — Binary flame images used for the construction and analysis of flame probability contour maps. (ZIP) [file pone.0336712.s003.zip › 10cm0.1/10_0.1144_cropped_cropped_adjusted.png]

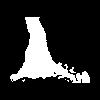

Supplement: S3 File — Binary flame images used for the construction and analysis of flame probability contour maps. (ZIP) [file pone.0336712.s003.zip › 10cm0.1/10_0.1145_cropped_cropped_adjusted.png]

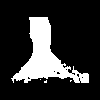

Supplement: S3 File — Binary flame images used for the construction and analysis of flame probability contour maps. (ZIP) [file pone.0336712.s003.zip › 10cm0.1/10_0.1146_cropped_cropped_adjusted.png]

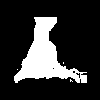

Supplement: S3 File — Binary flame images used for the construction and analysis of flame probability contour maps. (ZIP) [file pone.0336712.s003.zip › 10cm0.1/10_0.1147_cropped_cropped_adjusted.png]

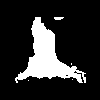

Supplement: S3 File — Binary flame images used for the construction and analysis of flame probability contour maps. (ZIP) [file pone.0336712.s003.zip › 10cm0.1/10_0.1148_cropped_cropped_adjusted.png]

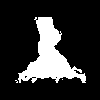

Supplement: S3 File — Binary flame images used for the construction and analysis of flame probability contour maps. (ZIP) [file pone.0336712.s003.zip › 10cm0.1/10_0.1149_cropped_cropped_adjusted.png]

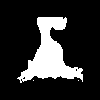

Supplement: S3 File — Binary flame images used for the construction and analysis of flame probability contour maps. (ZIP) [file pone.0336712.s003.zip › 10cm0.1/10_0.1150_cropped_cropped_adjusted.png]

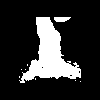

Supplement: S3 File — Binary flame images used for the construction and analysis of flame probability contour maps. (ZIP) [file pone.0336712.s003.zip › 10cm0.1/10_0.1151_cropped_cropped_adjusted.png]

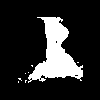

Supplement: S3 File — Binary flame images used for the construction and analysis of flame probability contour maps. (ZIP) [file pone.0336712.s003.zip › 10cm0.1/10_0.1152_cropped_cropped_adjusted.png]

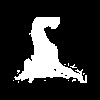

Supplement: S3 File — Binary flame images used for the construction and analysis of flame probability contour maps. (ZIP) [file pone.0336712.s003.zip › 10cm0.1/10_0.1153_cropped_cropped_adjusted.png]

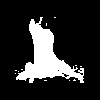

Supplement: S3 File — Binary flame images used for the construction and analysis of flame probability contour maps. (ZIP) [file pone.0336712.s003.zip › 10cm0.1/10_0.1154_cropped_cropped_adjusted.png]

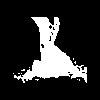

Supplement: S3 File — Binary flame images used for the construction and analysis of flame probability contour maps. (ZIP) [file pone.0336712.s003.zip › 10cm0.1/10_0.1155_cropped_cropped_adjusted.png]

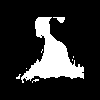

Supplement: S3 File — Binary flame images used for the construction and analysis of flame probability contour maps. (ZIP) [file pone.0336712.s003.zip › 10cm0.1/10_0.1156_cropped_cropped_adjusted.png]

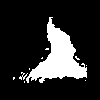

Supplement: S3 File — Binary flame images used for the construction and analysis of flame probability contour maps. (ZIP) [file pone.0336712.s003.zip › 10cm0.1/10_0.1157_cropped_cropped_adjusted.png]

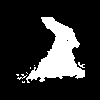

Supplement: S3 File — Binary flame images used for the construction and analysis of flame probability contour maps. (ZIP) [file pone.0336712.s003.zip › 10cm0.1/10_0.1158_cropped_cropped_adjusted.png]

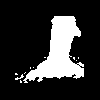

Supplement: S3 File — Binary flame images used for the construction and analysis of flame probability contour maps. (ZIP) [file pone.0336712.s003.zip › 10cm0.1/10_0.1159_cropped_cropped_adjusted.png]

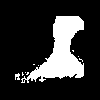

Supplement: S3 File — Binary flame images used for the construction and analysis of flame probability contour maps. (ZIP) [file pone.0336712.s003.zip › 10cm0.1/10_0.1160_cropped_cropped_adjusted.png]

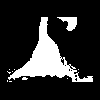

Supplement: S3 File — Binary flame images used for the construction and analysis of flame probability contour maps. (ZIP) [file pone.0336712.s003.zip › 10cm0.1/10_0.1161_cropped_cropped_adjusted.png]

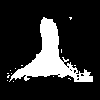

Supplement: S3 File — Binary flame images used for the construction and analysis of flame probability contour maps. (ZIP) [file pone.0336712.s003.zip › 10cm0.1/10_0.1162_cropped_cropped_adjusted.png]

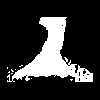

Supplement: S3 File — Binary flame images used for the construction and analysis of flame probability contour maps. (ZIP) [file pone.0336712.s003.zip › 10cm0.1/10_0.1163_cropped_cropped_adjusted.png]

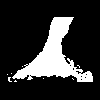

Supplement: S3 File — Binary flame images used for the construction and analysis of flame probability contour maps. (ZIP) [file pone.0336712.s003.zip › 10cm0.1/10_0.1164_cropped_cropped_adjusted.png]

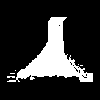

Supplement: S3 File — Binary flame images used for the construction and analysis of flame probability contour maps. (ZIP) [file pone.0336712.s003.zip › 10cm0.1/10_0.1165_cropped_cropped_adjusted.png]

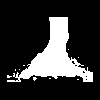

Supplement: S3 File — Binary flame images used for the construction and analysis of flame probability contour maps. (ZIP) [file pone.0336712.s003.zip › 10cm0.1/10_0.1166_cropped_cropped_adjusted.png]

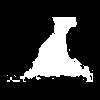

Supplement: S3 File — Binary flame images used for the construction and analysis of flame probability contour maps. (ZIP) [file pone.0336712.s003.zip › 10cm0.1/10_0.1167_cropped_cropped_adjusted.png]

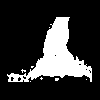

Supplement: S3 File — Binary flame images used for the construction and analysis of flame probability contour maps. (ZIP) [file pone.0336712.s003.zip › 10cm0.1/10_0.1168_cropped_cropped_adjusted.png]

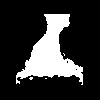

Supplement: S3 File — Binary flame images used for the construction and analysis of flame probability contour maps. (ZIP) [file pone.0336712.s003.zip › 10cm0.1/10_0.1168_cropped_cropped_cropped_adjusted.png]

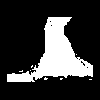

Supplement: S3 File — Binary flame images used for the construction and analysis of flame probability contour maps. (ZIP) [file pone.0336712.s003.zip › 10cm0.1/10_0.1169_cropped_cropped_adjusted.png]

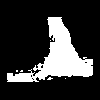

Supplement: S3 File — Binary flame images used for the construction and analysis of flame probability contour maps. (ZIP) [file pone.0336712.s003.zip › 10cm0.1/10_0.1170_cropped_cropped_adjusted.png]

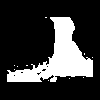

Supplement: S3 File — Binary flame images used for the construction and analysis of flame probability contour maps. (ZIP) [file pone.0336712.s003.zip › 10cm0.1/10_0.1171_cropped_cropped_adjusted.png]

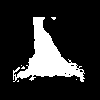

Supplement: S3 File — Binary flame images used for the construction and analysis of flame probability contour maps. (ZIP) [file pone.0336712.s003.zip › 10cm0.1/10_0.1172_cropped_cropped_adjusted.png]

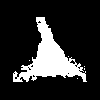

Supplement: S3 File — Binary flame images used for the construction and analysis of flame probability contour maps. (ZIP) [file pone.0336712.s003.zip › 10cm0.1/10_0.1173_cropped_cropped_adjusted.png]

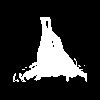

Supplement: S3 File — Binary flame images used for the construction and analysis of flame probability contour maps. (ZIP) [file pone.0336712.s003.zip › 10cm0.1/10_0.1174_cropped_cropped_adjusted.png]

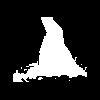

Supplement: S3 File — Binary flame images used for the construction and analysis of flame probability contour maps. (ZIP) [file pone.0336712.s003.zip › 10cm0.1/10_0.1175_cropped_cropped_adjusted.png]

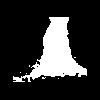

Supplement: S3 File — Binary flame images used for the construction and analysis of flame probability contour maps. (ZIP) [file pone.0336712.s003.zip › 10cm0.1/10_0.1176_cropped_cropped_adjusted.png]

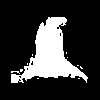

Supplement: S3 File — Binary flame images used for the construction and analysis of flame probability contour maps. (ZIP) [file pone.0336712.s003.zip › 10cm0.1/10_0.1177_cropped_cropped_adjusted.png]

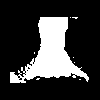

Supplement: S3 File — Binary flame images used for the construction and analysis of flame probability contour maps. (ZIP) [file pone.0336712.s003.zip › 10cm0.1/10_0.1178_cropped_cropped_adjusted.png]

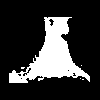

Supplement: S3 File — Binary flame images used for the construction and analysis of flame probability contour maps. (ZIP) [file pone.0336712.s003.zip › 10cm0.1/10_0.1179_cropped_cropped_adjusted.png]

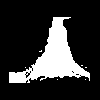

Supplement: S3 File — Binary flame images used for the construction and analysis of flame probability contour maps. (ZIP) [file pone.0336712.s003.zip › 10cm0.1/10_0.1180_cropped_cropped_adjusted.png]

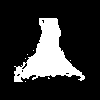

Supplement: S3 File — Binary flame images used for the construction and analysis of flame probability contour maps. (ZIP) [file pone.0336712.s003.zip › 10cm0.1/10_0.1181_cropped_cropped_adjusted.png]

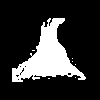

Supplement: S3 File — Binary flame images used for the construction and analysis of flame probability contour maps. (ZIP) [file pone.0336712.s003.zip › 10cm0.1/10_0.1182_cropped_cropped_adjusted.png]

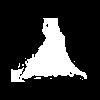

Supplement: S3 File — Binary flame images used for the construction and analysis of flame probability contour maps. (ZIP) [file pone.0336712.s003.zip › 10cm0.1/10_0.1183_cropped_cropped_adjusted.png]

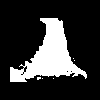

Supplement: S3 File — Binary flame images used for the construction and analysis of flame probability contour maps. (ZIP) [file pone.0336712.s003.zip › 10cm0.1/10_0.1184_cropped_cropped_adjusted.png]

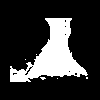

Supplement: S3 File — Binary flame images used for the construction and analysis of flame probability contour maps. (ZIP) [file pone.0336712.s003.zip › 10cm0.1/10_0.1185_cropped_cropped_adjusted.png]

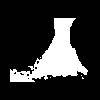

Supplement: S3 File — Binary flame images used for the construction and analysis of flame probability contour maps. (ZIP) [file pone.0336712.s003.zip › 10cm0.1/10_0.1186_cropped_cropped_adjusted.png]

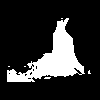

Supplement: S3 File — Binary flame images used for the construction and analysis of flame probability contour maps. (ZIP) [file pone.0336712.s003.zip › 10cm0.1/10_0.1187_cropped_cropped_adjusted.png]

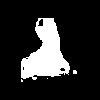

Supplement: S3 File — Binary flame images used for the construction and analysis of flame probability contour maps. (ZIP) [file pone.0336712.s003.zip › 10cm0.1/10_0.1188_cropped_cropped_adjusted.png]

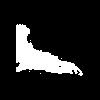

Supplement: S3 File — Binary flame images used for the construction and analysis of flame probability contour maps. (ZIP) [file pone.0336712.s003.zip › 10cm0.15/10_0.15001_cropped_cropped_cropped_adjusted.png]

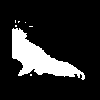

Supplement: S3 File — Binary flame images used for the construction and analysis of flame probability contour maps. (ZIP) [file pone.0336712.s003.zip › 10cm0.15/10_0.15002_cropped_cropped_cropped_adjusted.png]

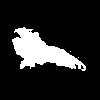

Supplement: S3 File — Binary flame images used for the construction and analysis of flame probability contour maps. (ZIP) [file pone.0336712.s003.zip › 10cm0.15/10_0.15003_cropped_cropped_cropped_adjusted.png]

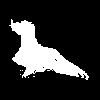

Supplement: S3 File — Binary flame images used for the construction and analysis of flame probability contour maps. (ZIP) [file pone.0336712.s003.zip › 10cm0.15/10_0.15004_cropped_cropped_cropped_adjusted.png]

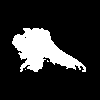

Supplement: S3 File — Binary flame images used for the construction and analysis of flame probability contour maps. (ZIP) [file pone.0336712.s003.zip › 10cm0.15/10_0.15005_cropped_cropped_cropped_adjusted.png]

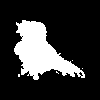

Supplement: S3 File — Binary flame images used for the construction and analysis of flame probability contour maps. (ZIP) [file pone.0336712.s003.zip › 10cm0.15/10_0.15006_cropped_cropped_cropped_adjusted.png]

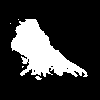

Supplement: S3 File — Binary flame images used for the construction and analysis of flame probability contour maps. (ZIP) [file pone.0336712.s003.zip › 10cm0.15/10_0.15007_cropped_cropped_cropped_adjusted.png]

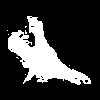

Supplement: S3 File — Binary flame images used for the construction and analysis of flame probability contour maps. (ZIP) [file pone.0336712.s003.zip › 10cm0.15/10_0.15008_cropped_cropped_cropped_adjusted.png]

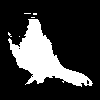

Supplement: S3 File — Binary flame images used for the construction and analysis of flame probability contour maps. (ZIP) [file pone.0336712.s003.zip › 10cm0.15/10_0.15009_cropped_cropped_cropped_adjusted.png]

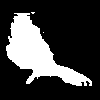

Supplement: S3 File — Binary flame images used for the construction and analysis of flame probability contour maps. (ZIP) [file pone.0336712.s003.zip › 10cm0.15/10_0.15010_cropped_cropped_cropped_adjusted.png]

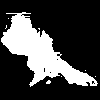

Supplement: S3 File — Binary flame images used for the construction and analysis of flame probability contour maps. (ZIP) [file pone.0336712.s003.zip › 10cm0.15/10_0.15011_cropped_cropped_cropped_adjusted.png]

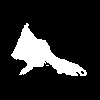

Supplement: S3 File — Binary flame images used for the construction and analysis of flame probability contour maps. (ZIP) [file pone.0336712.s003.zip › 10cm0.15/10_0.15012_cropped_cropped_cropped_adjusted.png]

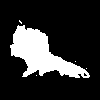

Supplement: S3 File — Binary flame images used for the construction and analysis of flame probability contour maps. (ZIP) [file pone.0336712.s003.zip › 10cm0.15/10_0.15013_cropped_cropped_cropped_adjusted.png]

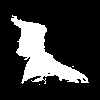

Supplement: S3 File — Binary flame images used for the construction and analysis of flame probability contour maps. (ZIP) [file pone.0336712.s003.zip › 10cm0.15/10_0.15014_cropped_cropped_cropped_adjusted.png]

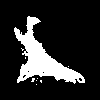

Supplement: S3 File — Binary flame images used for the construction and analysis of flame probability contour maps. (ZIP) [file pone.0336712.s003.zip › 10cm0.15/10_0.15015_cropped_cropped_cropped_adjusted.png]

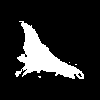

Supplement: S3 File — Binary flame images used for the construction and analysis of flame probability contour maps. (ZIP) [file pone.0336712.s003.zip › 10cm0.15/10_0.15016_cropped_cropped_cropped_adjusted.png]

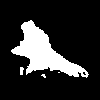

Supplement: S3 File — Binary flame images used for the construction and analysis of flame probability contour maps. (ZIP) [file pone.0336712.s003.zip › 10cm0.15/10_0.15017_cropped_cropped_cropped_adjusted.png]

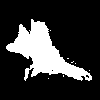

Supplement: S3 File — Binary flame images used for the construction and analysis of flame probability contour maps. (ZIP) [file pone.0336712.s003.zip › 10cm0.15/10_0.15018_cropped_cropped_cropped_adjusted.png]
