# Supplementary figures and images for: Experimental investigation of tunnel fire spread under moving fire source conditions (part 1 of 2)
Source: PLoS One. 2026 Feb 24;21(2):e0336712. doi: 10.1371/journal.pone.0336712 (PMC12931806; doi:10.1371/journal.pone.0336712)

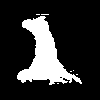

Supplement: S3 File — Binary flame images used for the construction and analysis of flame probability contour maps. (ZIP) [file pone.0336712.s003.zip › 10cm0.1/10_0.1001_cropped_cropped_cropped_adjusted.png]

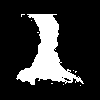

Supplement: S3 File — Binary flame images used for the construction and analysis of flame probability contour maps. (ZIP) [file pone.0336712.s003.zip › 10cm0.1/10_0.1002_cropped_cropped_cropped_adjusted.png]

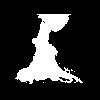

Supplement: S3 File — Binary flame images used for the construction and analysis of flame probability contour maps. (ZIP) [file pone.0336712.s003.zip › 10cm0.1/10_0.1003_cropped_cropped_cropped_adjusted.png]

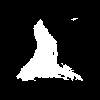

Supplement: S3 File — Binary flame images used for the construction and analysis of flame probability contour maps. (ZIP) [file pone.0336712.s003.zip › 10cm0.1/10_0.1004_cropped_cropped_cropped_adjusted.png]

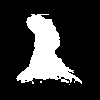

Supplement: S3 File — Binary flame images used for the construction and analysis of flame probability contour maps. (ZIP) [file pone.0336712.s003.zip › 10cm0.1/10_0.1005_cropped_cropped_cropped_adjusted.png]

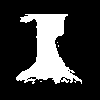

Supplement: S3 File — Binary flame images used for the construction and analysis of flame probability contour maps. (ZIP) [file pone.0336712.s003.zip › 10cm0.1/10_0.1006_cropped_cropped_cropped_adjusted.png]

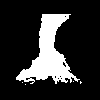

Supplement: S3 File — Binary flame images used for the construction and analysis of flame probability contour maps. (ZIP) [file pone.0336712.s003.zip › 10cm0.1/10_0.1007_cropped_cropped_cropped_adjusted.png]

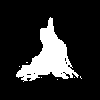

Supplement: S3 File — Binary flame images used for the construction and analysis of flame probability contour maps. (ZIP) [file pone.0336712.s003.zip › 10cm0.1/10_0.1008_cropped_cropped_cropped_adjusted.png]

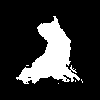

Supplement: S3 File — Binary flame images used for the construction and analysis of flame probability contour maps. (ZIP) [file pone.0336712.s003.zip › 10cm0.1/10_0.1009_cropped_cropped_cropped_adjusted.png]

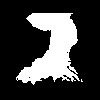

Supplement: S3 File — Binary flame images used for the construction and analysis of flame probability contour maps. (ZIP) [file pone.0336712.s003.zip › 10cm0.1/10_0.1010_cropped_cropped_cropped_adjusted.png]

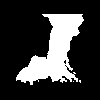

Supplement: S3 File — Binary flame images used for the construction and analysis of flame probability contour maps. (ZIP) [file pone.0336712.s003.zip › 10cm0.1/10_0.1011_cropped_cropped_cropped_adjusted.png]

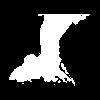

Supplement: S3 File — Binary flame images used for the construction and analysis of flame probability contour maps. (ZIP) [file pone.0336712.s003.zip › 10cm0.1/10_0.1012_cropped_cropped_cropped_adjusted.png]

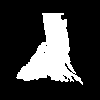

Supplement: S3 File — Binary flame images used for the construction and analysis of flame probability contour maps. (ZIP) [file pone.0336712.s003.zip › 10cm0.1/10_0.1013_cropped_cropped_cropped_adjusted.png]

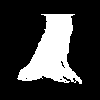

Supplement: S3 File — Binary flame images used for the construction and analysis of flame probability contour maps. (ZIP) [file pone.0336712.s003.zip › 10cm0.1/10_0.1014_cropped_cropped_cropped_adjusted.png]

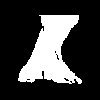

Supplement: S3 File — Binary flame images used for the construction and analysis of flame probability contour maps. (ZIP) [file pone.0336712.s003.zip › 10cm0.1/10_0.1015_cropped_cropped_cropped_adjusted.png]

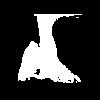

Supplement: S3 File — Binary flame images used for the construction and analysis of flame probability contour maps. (ZIP) [file pone.0336712.s003.zip › 10cm0.1/10_0.1016_cropped_cropped_cropped_adjusted.png]

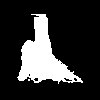

Supplement: S3 File — Binary flame images used for the construction and analysis of flame probability contour maps. (ZIP) [file pone.0336712.s003.zip › 10cm0.1/10_0.1017_cropped_cropped_cropped_adjusted.png]

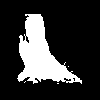

Supplement: S3 File — Binary flame images used for the construction and analysis of flame probability contour maps. (ZIP) [file pone.0336712.s003.zip › 10cm0.1/10_0.1018_cropped_cropped_cropped_adjusted.png]

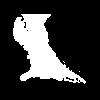

Supplement: S3 File — Binary flame images used for the construction and analysis of flame probability contour maps. (ZIP) [file pone.0336712.s003.zip › 10cm0.1/10_0.1019_cropped_cropped_cropped_adjusted.png]

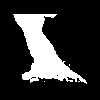

Supplement: S3 File — Binary flame images used for the construction and analysis of flame probability contour maps. (ZIP) [file pone.0336712.s003.zip › 10cm0.1/10_0.1020_cropped_cropped_cropped_adjusted.png]

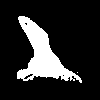

Supplement: S3 File — Binary flame images used for the construction and analysis of flame probability contour maps. (ZIP) [file pone.0336712.s003.zip › 10cm0.1/10_0.1021_cropped_cropped_cropped_adjusted.png]

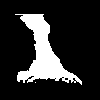

Supplement: S3 File — Binary flame images used for the construction and analysis of flame probability contour maps. (ZIP) [file pone.0336712.s003.zip › 10cm0.1/10_0.1022_cropped_cropped_cropped_adjusted.png]

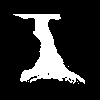

Supplement: S3 File — Binary flame images used for the construction and analysis of flame probability contour maps. (ZIP) [file pone.0336712.s003.zip › 10cm0.1/10_0.1023_cropped_cropped_cropped_adjusted.png]

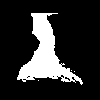

Supplement: S3 File — Binary flame images used for the construction and analysis of flame probability contour maps. (ZIP) [file pone.0336712.s003.zip › 10cm0.1/10_0.1024_cropped_cropped_cropped_adjusted.png]

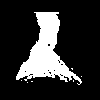

Supplement: S3 File — Binary flame images used for the construction and analysis of flame probability contour maps. (ZIP) [file pone.0336712.s003.zip › 10cm0.1/10_0.1025_cropped_cropped_cropped_adjusted.png]

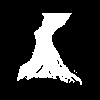

Supplement: S3 File — Binary flame images used for the construction and analysis of flame probability contour maps. (ZIP) [file pone.0336712.s003.zip › 10cm0.1/10_0.1026_cropped_cropped_cropped_adjusted.png]

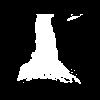

Supplement: S3 File — Binary flame images used for the construction and analysis of flame probability contour maps. (ZIP) [file pone.0336712.s003.zip › 10cm0.1/10_0.1027_cropped_cropped_cropped_adjusted.png]

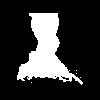

Supplement: S3 File — Binary flame images used for the construction and analysis of flame probability contour maps. (ZIP) [file pone.0336712.s003.zip › 10cm0.1/10_0.1028_cropped_cropped_cropped_adjusted.png]

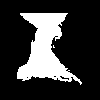

Supplement: S3 File — Binary flame images used for the construction and analysis of flame probability contour maps. (ZIP) [file pone.0336712.s003.zip › 10cm0.1/10_0.1029_cropped_cropped_cropped_adjusted.png]

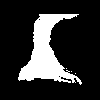

Supplement: S3 File — Binary flame images used for the construction and analysis of flame probability contour maps. (ZIP) [file pone.0336712.s003.zip › 10cm0.1/10_0.1030_cropped_cropped_cropped_adjusted.png]

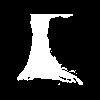

Supplement: S3 File — Binary flame images used for the construction and analysis of flame probability contour maps. (ZIP) [file pone.0336712.s003.zip › 10cm0.1/10_0.1031_cropped_cropped_cropped_adjusted.png]

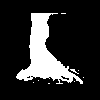

Supplement: S3 File — Binary flame images used for the construction and analysis of flame probability contour maps. (ZIP) [file pone.0336712.s003.zip › 10cm0.1/10_0.1032_cropped_cropped_cropped_adjusted.png]

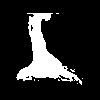

Supplement: S3 File — Binary flame images used for the construction and analysis of flame probability contour maps. (ZIP) [file pone.0336712.s003.zip › 10cm0.1/10_0.1033_cropped_cropped_cropped_adjusted.png]

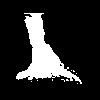

Supplement: S3 File — Binary flame images used for the construction and analysis of flame probability contour maps. (ZIP) [file pone.0336712.s003.zip › 10cm0.1/10_0.1034_cropped_cropped_cropped_adjusted.png]

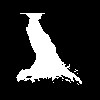

Supplement: S3 File — Binary flame images used for the construction and analysis of flame probability contour maps. (ZIP) [file pone.0336712.s003.zip › 10cm0.1/10_0.1035_cropped_cropped_cropped_adjusted.png]

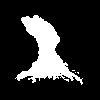

Supplement: S3 File — Binary flame images used for the construction and analysis of flame probability contour maps. (ZIP) [file pone.0336712.s003.zip › 10cm0.1/10_0.1036_cropped_cropped_cropped_adjusted.png]

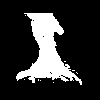

Supplement: S3 File — Binary flame images used for the construction and analysis of flame probability contour maps. (ZIP) [file pone.0336712.s003.zip › 10cm0.1/10_0.1037_cropped_cropped_cropped_adjusted.png]

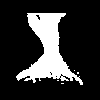

Supplement: S3 File — Binary flame images used for the construction and analysis of flame probability contour maps. (ZIP) [file pone.0336712.s003.zip › 10cm0.1/10_0.1038_cropped_cropped_cropped_adjusted.png]

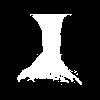

Supplement: S3 File — Binary flame images used for the construction and analysis of flame probability contour maps. (ZIP) [file pone.0336712.s003.zip › 10cm0.1/10_0.1039_cropped_cropped_cropped_adjusted.png]

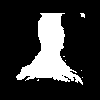

Supplement: S3 File — Binary flame images used for the construction and analysis of flame probability contour maps. (ZIP) [file pone.0336712.s003.zip › 10cm0.1/10_0.1040_cropped_cropped_cropped_adjusted.png]

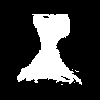

Supplement: S3 File — Binary flame images used for the construction and analysis of flame probability contour maps. (ZIP) [file pone.0336712.s003.zip › 10cm0.1/10_0.1041_cropped_cropped_cropped_adjusted.png]

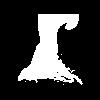

Supplement: S3 File — Binary flame images used for the construction and analysis of flame probability contour maps. (ZIP) [file pone.0336712.s003.zip › 10cm0.1/10_0.1042_cropped_cropped_cropped_adjusted.png]

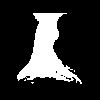

Supplement: S3 File — Binary flame images used for the construction and analysis of flame probability contour maps. (ZIP) [file pone.0336712.s003.zip › 10cm0.1/10_0.1043_cropped_cropped_cropped_adjusted.png]

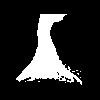

Supplement: S3 File — Binary flame images used for the construction and analysis of flame probability contour maps. (ZIP) [file pone.0336712.s003.zip › 10cm0.1/10_0.1044_cropped_cropped_cropped_adjusted.png]

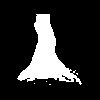

Supplement: S3 File — Binary flame images used for the construction and analysis of flame probability contour maps. (ZIP) [file pone.0336712.s003.zip › 10cm0.1/10_0.1045_cropped_cropped_cropped_adjusted.png]

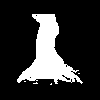

Supplement: S3 File — Binary flame images used for the construction and analysis of flame probability contour maps. (ZIP) [file pone.0336712.s003.zip › 10cm0.1/10_0.1046_cropped_cropped_cropped_adjusted.png]

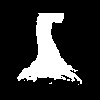

Supplement: S3 File — Binary flame images used for the construction and analysis of flame probability contour maps. (ZIP) [file pone.0336712.s003.zip › 10cm0.1/10_0.1047_cropped_cropped_cropped_adjusted.png]

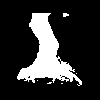

Supplement: S3 File — Binary flame images used for the construction and analysis of flame probability contour maps. (ZIP) [file pone.0336712.s003.zip › 10cm0.1/10_0.1048_cropped_cropped_cropped_adjusted.png]

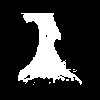

Supplement: S3 File — Binary flame images used for the construction and analysis of flame probability contour maps. (ZIP) [file pone.0336712.s003.zip › 10cm0.1/10_0.1049_cropped_cropped_cropped_adjusted.png]

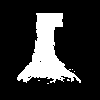

Supplement: S3 File — Binary flame images used for the construction and analysis of flame probability contour maps. (ZIP) [file pone.0336712.s003.zip › 10cm0.1/10_0.1050_cropped_cropped_cropped_adjusted.png]

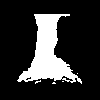

Supplement: S3 File — Binary flame images used for the construction and analysis of flame probability contour maps. (ZIP) [file pone.0336712.s003.zip › 10cm0.1/10_0.1051_cropped_cropped_cropped_adjusted.png]

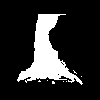

Supplement: S3 File — Binary flame images used for the construction and analysis of flame probability contour maps. (ZIP) [file pone.0336712.s003.zip › 10cm0.1/10_0.1052_cropped_cropped_cropped_adjusted.png]

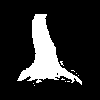

Supplement: S3 File — Binary flame images used for the construction and analysis of flame probability contour maps. (ZIP) [file pone.0336712.s003.zip › 10cm0.1/10_0.1053_cropped_cropped_cropped_adjusted.png]

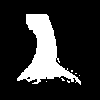

Supplement: S3 File — Binary flame images used for the construction and analysis of flame probability contour maps. (ZIP) [file pone.0336712.s003.zip › 10cm0.1/10_0.1054_cropped_cropped_cropped_adjusted.png]

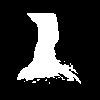

Supplement: S3 File — Binary flame images used for the construction and analysis of flame probability contour maps. (ZIP) [file pone.0336712.s003.zip › 10cm0.1/10_0.1055_cropped_cropped_cropped_adjusted.png]

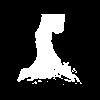

Supplement: S3 File — Binary flame images used for the construction and analysis of flame probability contour maps. (ZIP) [file pone.0336712.s003.zip › 10cm0.1/10_0.1056_cropped_cropped_cropped_adjusted.png]

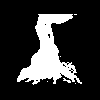

Supplement: S3 File — Binary flame images used for the construction and analysis of flame probability contour maps. (ZIP) [file pone.0336712.s003.zip › 10cm0.1/10_0.1057_cropped_cropped_cropped_adjusted.png]

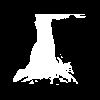

Supplement: S3 File — Binary flame images used for the construction and analysis of flame probability contour maps. (ZIP) [file pone.0336712.s003.zip › 10cm0.1/10_0.1058_cropped_cropped_cropped_adjusted.png]

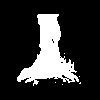

Supplement: S3 File — Binary flame images used for the construction and analysis of flame probability contour maps. (ZIP) [file pone.0336712.s003.zip › 10cm0.1/10_0.1059_cropped_cropped_cropped_adjusted.png]

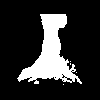

Supplement: S3 File — Binary flame images used for the construction and analysis of flame probability contour maps. (ZIP) [file pone.0336712.s003.zip › 10cm0.1/10_0.1060_cropped_cropped_cropped_adjusted.png]

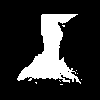

Supplement: S3 File — Binary flame images used for the construction and analysis of flame probability contour maps. (ZIP) [file pone.0336712.s003.zip › 10cm0.1/10_0.1061_cropped_cropped_cropped_adjusted.png]

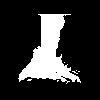

Supplement: S3 File — Binary flame images used for the construction and analysis of flame probability contour maps. (ZIP) [file pone.0336712.s003.zip › 10cm0.1/10_0.1062_cropped_cropped_cropped_adjusted.png]

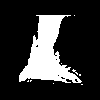

Supplement: S3 File — Binary flame images used for the construction and analysis of flame probability contour maps. (ZIP) [file pone.0336712.s003.zip › 10cm0.1/10_0.1063_cropped_cropped_cropped_adjusted.png]

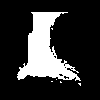

Supplement: S3 File — Binary flame images used for the construction and analysis of flame probability contour maps. (ZIP) [file pone.0336712.s003.zip › 10cm0.1/10_0.1064_cropped_cropped_cropped_adjusted.png]

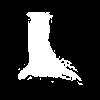

Supplement: S3 File — Binary flame images used for the construction and analysis of flame probability contour maps. (ZIP) [file pone.0336712.s003.zip › 10cm0.1/10_0.1065_cropped_cropped_cropped_adjusted.png]

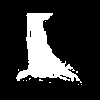

Supplement: S3 File — Binary flame images used for the construction and analysis of flame probability contour maps. (ZIP) [file pone.0336712.s003.zip › 10cm0.1/10_0.1066_cropped_cropped_cropped_adjusted.png]

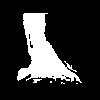

Supplement: S3 File — Binary flame images used for the construction and analysis of flame probability contour maps. (ZIP) [file pone.0336712.s003.zip › 10cm0.1/10_0.1067_cropped_cropped_cropped_adjusted.png]

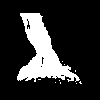

Supplement: S3 File — Binary flame images used for the construction and analysis of flame probability contour maps. (ZIP) [file pone.0336712.s003.zip › 10cm0.1/10_0.1068_cropped_cropped_cropped_adjusted.png]

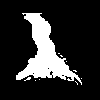

Supplement: S3 File — Binary flame images used for the construction and analysis of flame probability contour maps. (ZIP) [file pone.0336712.s003.zip › 10cm0.1/10_0.1069_cropped_cropped_cropped_adjusted.png]

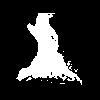

Supplement: S3 File — Binary flame images used for the construction and analysis of flame probability contour maps. (ZIP) [file pone.0336712.s003.zip › 10cm0.1/10_0.1070_cropped_cropped_cropped_adjusted.png]

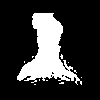

Supplement: S3 File — Binary flame images used for the construction and analysis of flame probability contour maps. (ZIP) [file pone.0336712.s003.zip › 10cm0.1/10_0.1071_cropped_cropped_cropped_adjusted.png]

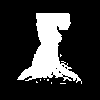

Supplement: S3 File — Binary flame images used for the construction and analysis of flame probability contour maps. (ZIP) [file pone.0336712.s003.zip › 10cm0.1/10_0.1072_cropped_cropped_cropped_adjusted.png]

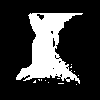

Supplement: S3 File — Binary flame images used for the construction and analysis of flame probability contour maps. (ZIP) [file pone.0336712.s003.zip › 10cm0.1/10_0.1073_cropped_cropped_cropped_adjusted.png]

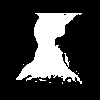

Supplement: S3 File — Binary flame images used for the construction and analysis of flame probability contour maps. (ZIP) [file pone.0336712.s003.zip › 10cm0.1/10_0.1074_cropped_cropped_cropped_adjusted.png]

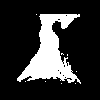

Supplement: S3 File — Binary flame images used for the construction and analysis of flame probability contour maps. (ZIP) [file pone.0336712.s003.zip › 10cm0.1/10_0.1075_cropped_cropped_cropped_adjusted.png]

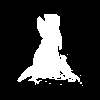

Supplement: S3 File — Binary flame images used for the construction and analysis of flame probability contour maps. (ZIP) [file pone.0336712.s003.zip › 10cm0.1/10_0.1076_cropped_cropped_cropped_adjusted.png]

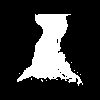

Supplement: S3 File — Binary flame images used for the construction and analysis of flame probability contour maps. (ZIP) [file pone.0336712.s003.zip › 10cm0.1/10_0.1077_cropped_cropped_cropped_adjusted.png]

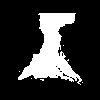

Supplement: S3 File — Binary flame images used for the construction and analysis of flame probability contour maps. (ZIP) [file pone.0336712.s003.zip › 10cm0.1/10_0.1078_cropped_cropped_cropped_adjusted.png]

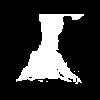

Supplement: S3 File — Binary flame images used for the construction and analysis of flame probability contour maps. (ZIP) [file pone.0336712.s003.zip › 10cm0.1/10_0.1079_cropped_cropped_cropped_adjusted.png]

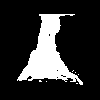

Supplement: S3 File — Binary flame images used for the construction and analysis of flame probability contour maps. (ZIP) [file pone.0336712.s003.zip › 10cm0.1/10_0.1080_cropped_cropped_cropped_adjusted.png]

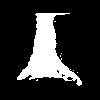

Supplement: S3 File — Binary flame images used for the construction and analysis of flame probability contour maps. (ZIP) [file pone.0336712.s003.zip › 10cm0.1/10_0.1081_cropped_cropped_cropped_adjusted.png]

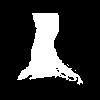

Supplement: S3 File — Binary flame images used for the construction and analysis of flame probability contour maps. (ZIP) [file pone.0336712.s003.zip › 10cm0.1/10_0.1082_cropped_cropped_cropped_adjusted.png]

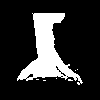

Supplement: S3 File — Binary flame images used for the construction and analysis of flame probability contour maps. (ZIP) [file pone.0336712.s003.zip › 10cm0.1/10_0.1083_cropped_cropped_cropped_adjusted.png]

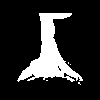

Supplement: S3 File — Binary flame images used for the construction and analysis of flame probability contour maps. (ZIP) [file pone.0336712.s003.zip › 10cm0.1/10_0.1084_cropped_cropped_cropped_adjusted.png]

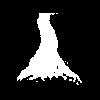

Supplement: S3 File — Binary flame images used for the construction and analysis of flame probability contour maps. (ZIP) [file pone.0336712.s003.zip › 10cm0.1/10_0.1085_cropped_cropped_cropped_adjusted.png]

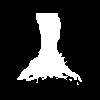

Supplement: S3 File — Binary flame images used for the construction and analysis of flame probability contour maps. (ZIP) [file pone.0336712.s003.zip › 10cm0.1/10_0.1086_cropped_cropped_cropped_adjusted.png]

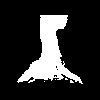

Supplement: S3 File — Binary flame images used for the construction and analysis of flame probability contour maps. (ZIP) [file pone.0336712.s003.zip › 10cm0.1/10_0.1087_cropped_cropped_cropped_adjusted.png]

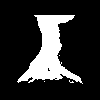

Supplement: S3 File — Binary flame images used for the construction and analysis of flame probability contour maps. (ZIP) [file pone.0336712.s003.zip › 10cm0.1/10_0.1088_cropped_cropped_cropped_adjusted.png]

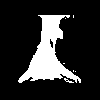

Supplement: S3 File — Binary flame images used for the construction and analysis of flame probability contour maps. (ZIP) [file pone.0336712.s003.zip › 10cm0.1/10_0.1089_cropped_cropped_cropped_adjusted.png]

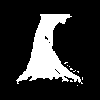

Supplement: S3 File — Binary flame images used for the construction and analysis of flame probability contour maps. (ZIP) [file pone.0336712.s003.zip › 10cm0.1/10_0.1090_cropped_cropped_cropped_adjusted.png]

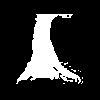

Supplement: S3 File — Binary flame images used for the construction and analysis of flame probability contour maps. (ZIP) [file pone.0336712.s003.zip › 10cm0.1/10_0.1091_cropped_cropped_cropped_adjusted.png]

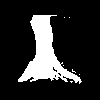

Supplement: S3 File — Binary flame images used for the construction and analysis of flame probability contour maps. (ZIP) [file pone.0336712.s003.zip › 10cm0.1/10_0.1092_cropped_cropped_cropped_adjusted.png]

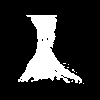

Supplement: S3 File — Binary flame images used for the construction and analysis of flame probability contour maps. (ZIP) [file pone.0336712.s003.zip › 10cm0.1/10_0.1093_cropped_cropped_cropped_adjusted.png]

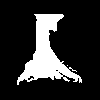

Supplement: S3 File — Binary flame images used for the construction and analysis of flame probability contour maps. (ZIP) [file pone.0336712.s003.zip › 10cm0.1/10_0.1094_cropped_cropped_cropped_adjusted.png]

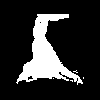

Supplement: S3 File — Binary flame images used for the construction and analysis of flame probability contour maps. (ZIP) [file pone.0336712.s003.zip › 10cm0.1/10_0.1095_cropped_cropped_cropped_adjusted.png]

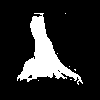

Supplement: S3 File — Binary flame images used for the construction and analysis of flame probability contour maps. (ZIP) [file pone.0336712.s003.zip › 10cm0.1/10_0.1096_cropped_cropped_cropped_adjusted.png]

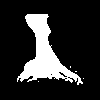

Supplement: S3 File — Binary flame images used for the construction and analysis of flame probability contour maps. (ZIP) [file pone.0336712.s003.zip › 10cm0.1/10_0.1097_cropped_cropped_cropped_adjusted.png]

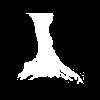

Supplement: S3 File — Binary flame images used for the construction and analysis of flame probability contour maps. (ZIP) [file pone.0336712.s003.zip › 10cm0.1/10_0.1098_cropped_cropped_cropped_adjusted.png]

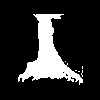

Supplement: S3 File — Binary flame images used for the construction and analysis of flame probability contour maps. (ZIP) [file pone.0336712.s003.zip › 10cm0.1/10_0.1099_cropped_cropped_cropped_adjusted.png]

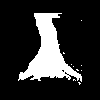

Supplement: S3 File — Binary flame images used for the construction and analysis of flame probability contour maps. (ZIP) [file pone.0336712.s003.zip › 10cm0.1/10_0.1100_cropped_cropped_cropped_adjusted.png]
